# Image-based computational quantification and visualization of genetic alterations and tumour heterogeneity

**DOI:** 10.1038/srep24146

**Published:** 2016-04-07

**Authors:** Qing Zhong, Jan H. Rüschoff, Tiannan Guo, Maria Gabrani, Peter J. Schüffler, Markus Rechsteiner, Yansheng Liu, Thomas J. Fuchs, Niels J. Rupp, Christian Fankhauser, Joachim M. Buhmann, Sven Perner, Cédric Poyet, Miriam Blattner, Davide Soldini, Holger Moch, Mark A. Rubin, Aurelia Noske, Josef Rüschoff, Michael C. Haffner, Wolfram Jochum, Peter J. Wild

**Affiliations:** 1 Institute of Surgical Pathology, University Hospital Zurich, Zurich, Switzerland; 2 Institute of Molecular Systems Biology, ETH, Zurich, Switzerland; 3 Zurich Labouratory, IBM Research-Zurich, Rueschlikon, Switzerland; 4 Department of Computer Science, ETH, Zurich, Switzerland; 5 Memorial Sloan Kettering Cancer Center, New York, NY, USA; 6 Department of Prostate Cancer Research, Institute of Pathology, University Hospital of Bonn, Bonn, Germany; 7 Department of Urology, University Hospital Zurich, Zurich, Switzerland.; 8 Institute for Precision Medicine and Department of Pathology and Laboratory Medicine, Weill Medical College of Cornell University and New York-Presbyterian Hospital, New York, NY, USA; 9 Targos Molecular Pathology, Pathology Nordhessen, Kassel, Germany; 10 Sidney Kimmel Comprehensive Cancer Center, Johns Hopkins University, Baltimore, Maryland, USA; 11 Institute of Pathology, Cantonal Hospital St. Gallen, St. Gallen, Switzerland

## Abstract

Recent large-scale genome analyses of human tissue samples have uncovered a high degree of genetic alterations and tumour heterogeneity in most tumour entities, independent of morphological phenotypes and histopathological characteristics. Assessment of genetic copy-number variation (CNV) and tumour heterogeneity by fluorescence *in situ* hybridization (ISH) provides additional tissue morphology at single-cell resolution, but it is labour intensive with limited throughput and high inter-observer variability. We present an integrative method combining bright-field dual-colour chromogenic and silver ISH assays with an image-based computational workflow (ISHProfiler), for accurate detection of molecular signals, high-throughput evaluation of CNV, expressive visualization of multi-level heterogeneity (cellular, inter- and intra-tumour heterogeneity), and objective quantification of heterogeneous genetic deletions (*PTEN*) and amplifications (19q12, *HER2*) in diverse human tumours (prostate, endometrial, ovarian and gastric), using various tissue sizes and different scanners, with unprecedented throughput and reproducibility.

Next-generation sequencing (NGS) studies of various tumours have uncovered multiple genetic abnormalities and extensive tumour heterogeneity, demonstrating its substantial impact on cancer treatment and personalized medicine^1^,^2^,^3^,^4^. Yet, NGS studies often preclude potential integrative analysis with the corresponding morphological phenotype, thus losing important topological information of tissue architecture. Moreover, genome sequencing has usually been performed on bulk tumour specimens, which often fails to identify minor sub-clones and predict whether the mutations occur in the same or in different cells. Although modern technologies have solved these problems by sequencing single cancer cells, their implementations are still inapplicable for large research cohorts^5^.

Fluorescence *in situ* hybridization (FISH) is a well-established method to measure genetic variations along with tissue morphology at cellular resolution^6^. Nevertheless, the requirement of fluorescence microscopy, fading fluorescent signals, and the subjective interpretation of copy number variation (CNV)^7^,^8^,^9^, impede throughput, induce biased assessment among different investigators^10^,^11^,^12^, and hinder a systematic quantification of tumour heterogeneity.

To overcome these limitations, we established a dual-colour chromogenic and silver *in situ* hybridization (DISH) assay to permit bright-field evaluation of morphological details, developed an image-based computational workflow (ISHProfiler) to detect CNV at single-cell level, and proposed a statistical analysis to quantify tumour heterogeneity for a variety of genes across several cancer entities on tissue microarrays (TMAs) and whole-slide images with accompanying visualization tools. Here, we demonstrate the versatility of ISHProfiler and provide proof-of-principle evidence for its application to precision medicine for objective patient stratification.

## Results

### Reference data and a novel scoring method

**Figure 1:**
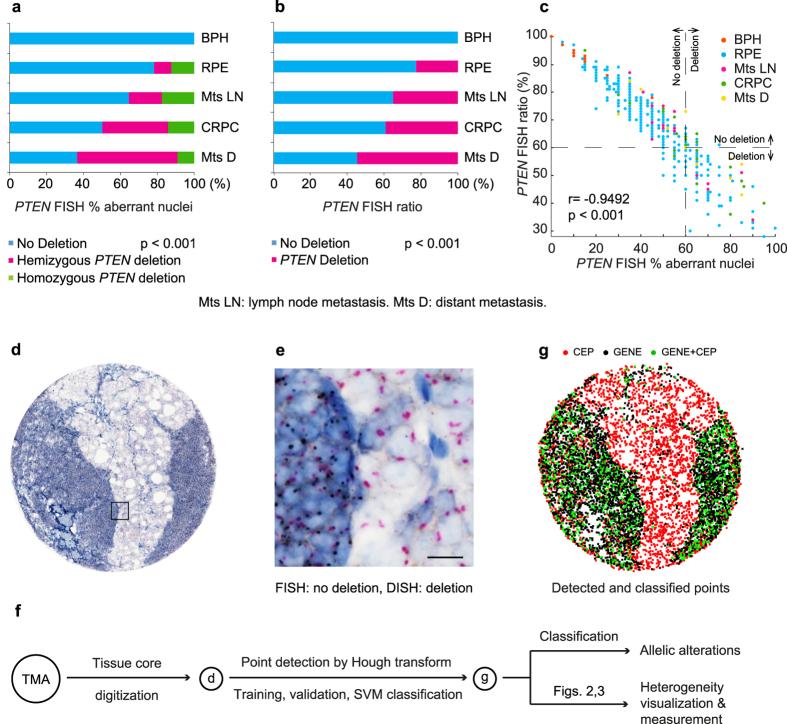
FISH, DISH and ISHProfiler for quantification and visualization of genetic alterations. (**a**) *PTEN* loss assessed by FISH on FFPE tissue sections as an independent negative prognostic marker for PC. Cumulative bar charts showing the association of *PTEN* deletion based on the percentage of aberrant nuclei with different prostate tissue types. *P* value was calculated with the two-side Fisher’s exact test. (**b**) Cumulative bar charts for the ratio. (**c**) Scatterplot of the percentage aberrant nuclei against the ratio, colour-coded by tissue types. The threshold was set to 60% for both scoring methods. Linear correlation revealed *r* = −0.9492 and *P* < 0.001. (**d**) Example of a PC lymph node metastasis showing cellular heterogeneity for *PTEN* status. (**e**) Zoomed image showing PC with *PTEN* deletion (right side) and lymph node structures without *PTEN* deletion (left side). Black signal: *PTEN* gene; red signal: CEP10. Scale bar, 10 μm. (**f**) Computational workflow ISHProfiler. Circled letters correspond to the respective results shown in (**d,g**). (**g**) Detected and classified *PTEN* gene and CEP points are displayed as a signal colour map. Black signal: *PTEN* gene; red signal: CEP10; green signal: *PTEN*+CEP10.

As reference data for introducing a novel scoring method to estimate genetic alterations, we used a TMA of human prostate cancer (PC) hybridized with FISH probes for the tumour suppressor gene *phosphatase and tensin homolog* (*PTEN*) and the corresponding centromeric probe (CEP) of chromosome 10. In contrast to the conventional scoring approach based on estimation of the percentage of aberrant nuclei^9^,^12^, the new score represents the ratio of *PTEN* to CEP10 of a given tumour region without the requirement for recognizing distinct nuclei^13^,^14^, therefore diminishing the signal loss effect caused by cutting artifacts^15^. To benchmark this ratio score for the estimation of *PTEN* deletion in PC, we used 424 benign and malignant prostate formalin-fixed paraffin-embedded (FFPE) tissue samples, consisting of 339 radical prostatectomy (RPE) specimens, 28 castration resistant prostate cancers (CRPCs), 17 lymph node metastases, 11 distant metastases, and 29 benign prostatic hyperplasias (BPHs). *PTEN* homozygous and hemizygous deletion, based on the manual counting of FISH signals and classification at the threshold of 60%^9^ for both scoring methods, indicated significant associations (*P* < 0.001) of *PTEN* deletion with different tissue types (Fig. 1a,b). Equivalence of both scores was further confirmed by multiple comparisons with clinico-pathologic, immunologic, and genetic features of patients receiving RPE (Supplementary Fig. S1 and Supplementary Table S1), by linear correlation (*r* = −0.9492, *P* < 0.001; Fig. 1c), and by analysis of overall (log-rank *P* = 0.017 and 0.003), disease-specific (log-rank *P* = 0.009 and 0.005), as well as recurrence-free (log-rank *P* = 0.209 and 0.387) survival (Supplementary Fig. S2). Last, univariate and multivariate Cox regression revealed that *PTEN* deletion estimated by the ratio score is a strong prognostic factor for overall survival in PC (*P* = 0.0208, hazard ratio = 2.0, 95% confidence interval (CI) [1.11–3.60]); Supplementary Fig. S3).

### PTEN DISH assay versus FISH assay

We established a *PTEN* DISH assay to streamline the detection of *PTEN* deletion in a representative subset of 71 tissue samples, providing permanent staining and detailed histological morphology compared with FISH (Supplementary Fig. S4). These tissue cores were analysed by both *PTEN* DISH and FISH assays using the ratio score: 38 primary acinar adenocarcinomas from RPE specimens, ten CRPCs, six PC lymph node metastases, one distant metastasis, and 16 BPHs. DISH assessment by manual counting and ratio scoring of *PTEN* and CEP10 signals was highly concordant with that of FISH (classification accuracy 94.4%, sensitivity 92.3%, and specificity 94.8%; Supplementary Table S2). The sole false negative case (FISH: deletion, DISH: no deletion; Fig. 1d, and the zoomed version: Fig. 1e) and three false positive cases (Supplementary Fig. S5) strongly supported the notion that misclassifications were attributed to cellular heterogeneity (different cell types within a tissue core), intra-tumour heterogeneity (ITH), and inter-observer variability (two pathologists) rather than to the malperformance of the DISH assay. Moreover, manual evaluation of DISH is labour intensive and becomes infeasible for large-scale cohorts. These problems emphasized the need for accurate detection of molecular signals, fast CNV assessment, and quantitative measurement of tumour heterogeneity.

### An image-based computational workflow - ISHProfiler

To automate DISH analysis and produce unbiased assessment of CNVs, we developed an image-based computational workflow (Fig. 1f) for ISH assays (ISHProfiler), which has been integrated into the open source software TMARKER^16^. ISHProfiler uses supervised machine learning and statistical methods to generate computational models of CNV based on the classification of detected molecular signals, without relying on computationally intensive algorithms for single-cell recognition^13^,^14^. The workflow consists of three major algorithmic steps: First, each tissue was digitized, pre-processed, and resized. Second, DISH signals (1,000 to 5,000 signals per tissue core, and more than a million for a whole slide image) were detected by the circular Hough transform^17^. Third, a support vector machine (SVM) model^18^ was trained and 5-fold cross validated on the basis of an independent training set of *PTEN* DISH signals with expert annotations (Supplementary Fig. S6). The final model was used to classify the signals into five classes: *PTEN*, CEP10, mixed class *PTEN*+CEP10, background noise and cell stains; about 30% of signals were classified as *PTEN* or CEP10. Analogous to the novel ratio scoring method, the global ratio was defined as the division of all *PTEN* by all CEP10 signals in a single tissue core.

Using all 71 global ratios as prediction scores, receiver operating characteristic (ROC) analysis yielded a large area under the curve value of 0.99 (95% CI [0.96–1.00]), and found the optimal dichotomization threshold at 84% with a total accuracy of 97.2% (Supplementary Fig. S7 and Supplementary Table S3), enabling objective determination of the threshold that was previously determined in an empirical manner^9^. Moreover, the three-step algorithm is an independent process, thus loop iterations over each tissue or sub-image of a large whole slide can be executed in parallel. Thus, ISHProfiler achieved a classification accuracy similar to that of manual assessment, while the evaluation time was tremendously reduced, outperforming manual assessment by at least four orders of magnitude.

### ISHProfiler for visualization and quantification of multi-level heterogeneity

**Figure 2:**
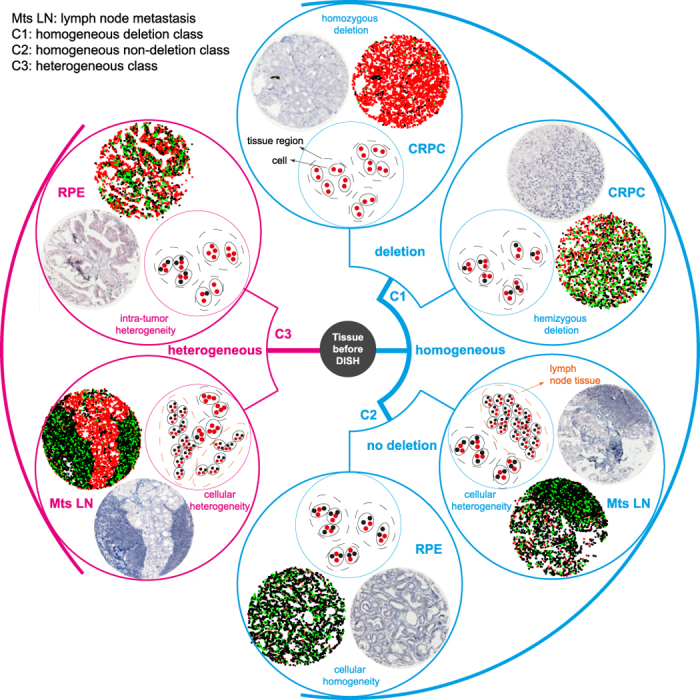
Visualization of multi-level heterogeneity by ISHProfiler. Heterogeneity of *PTEN* CNV of a given tissue core can be visually classified into two major groups (homogeneous and heterogeneous classes), three subclasses (C1 and C2: homogeneous deletion and non-deletion, and C3: heterogeneous class) or six subgroups (homozygous deletion, hemizygous deletion, cellular homogeneity, cellular heterogeneity with either homogeneous or heterogeneous genetic status, and ITH), which are illustrated as original tissue core image, signal colour map, and sketch.

Deletion of *PTEN* has been shown to be heterogeneous and subclonal^19^, and is associated with tumour progression^9^,^20^,^21^,^22^. Therefore, analysis of *PTEN* status provides an appropriate means for investigating tumour heterogeneity and subclonal evolution. Dichotomization of *PTEN* status into deletion and non-deletion using an empirical^9^ or single-valued threshold is arbitrary, particularly in the presence of tumour heterogeneity. We applied ISHProfiler to the 71 tissue cores and generated respective signal colour maps, in which *PTEN* and CEP10 were illustrated as coloured squares (Fig. 1g). The descriptive colour pattern allowed for a straightforward visual categorization of these tissues into two major classes of homogenous and heterogeneous events, three subclasses and six prototypes (Fig. 2). While the three subclasses further divided the homogenous events into deletion and non-deletion, the six prototypes differentiated subtypes such as homozygous deletion, hemizygous deletion, cellular homogeneity, cellular heterogeneity with either homogeneous or heterogeneous genetic status, and ITH.

To quantify cellular, inter-, and intra-tumour heterogeneity among and within individual tissue cores, we extended ISHProfiler by incorporating the randomized local ratio (RLR) and the randomized local density (RLD) into the analysis. While the global ratio characterized each tissue core as a single number, the RLR and RLD measured the distribution of local ratios and signal densities that were computed in the respective vicinity of cells at random (Supplementary Fig. S8), providing a plausible quantification of heterogeneity at multiple levels. We then constructed multivariate features by extracting statistics, such as central tendency and dispersion from the distributions of all RLR and RLD, to which we performed principal component analysis (PCA) for dimensionality reduction.

**Figure 3:**
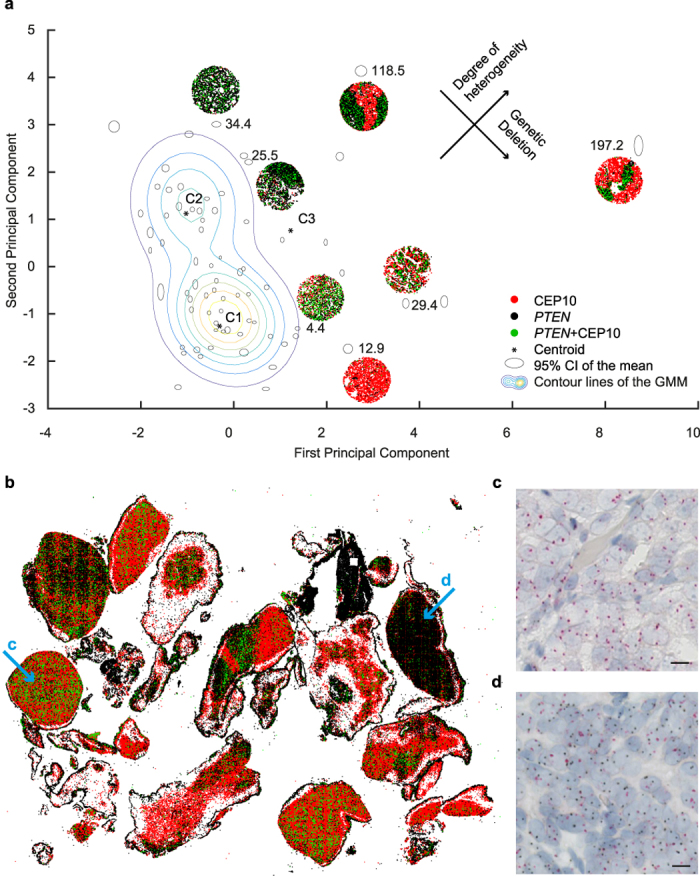
Quantification of tumour heterogeneity by ISHProfiler. (**a**) Tissue core distribution in the two-dimensional principal component analysis subspace (*n* = 71), superimposed with selected signal colour maps shown in Fig. 2. Ellipses refer to individual tissue cores. The axes of the ellipses indicate the 95% CI of an experiment with a total of *n* = 100 repetitions by varying the number of random points from 201 to 300. The number is the Mahalanobis distance of each point to the respective centroids that are illustrated as stars. (**b**) A signal colour map generated from a whole slide image of CRPC shows detected and classified *PTEN* and CEP10 signals. (**c,d**) Zoomed image of areas marked with arrows in (**b)**. Scale bar, 10 μm.

We further applied a probabilistic model by Gaussian mixture modelling (GMM) to assign the 71 tissue cores into three clusters approximating the three subclasses defined in (Fig. 2), and calculated the Mahalanobis distances of each point to the centroid (the mean of a designated distribution) of the homogeneous deletion class. The Mahalanobis distance measures the distance (the amount of standard deviations) between a given tissue core and the centroid. In the two-dimensional PCA subspace, tissues with both cellular and intra-tumour heterogeneity lay farthest from the centroids of the two homogeneous classes (C1 and C2 in Fig. 3a), followed by tissues with ITH, while tissues with homozygous and hemizygous deletion, and non-deletion were placed in an identical order as in (Fig. 2). Thus, the top-left to bottom-right diagonals can be interpreted as the progression of *PTEN* deletion and the anti-diagonal as the degree of heterogeneity for *PTEN* status. In addition, inter-tumour heterogeneity was exemplified by the pairwise distance of each tissue core, and cellular heterogeneity by the discrepant position of the two lymph node metastases (top centre and far right spot; Fig. 3a) that exhibited different amounts of lymphoid tissue.

To examine the robustness of the two random methods, we varied the number of random points for each core from 201 to 300, recalculated RLR and RLD, performed PCA, and applied GMM. Each core was then represented by an ellipse (Fig. 3a) with its centre as the mean of the 100 experiments and the axes as the 95% CI. This perturbation experiment suggested that ISHProfiler is not only robust but also insensitive to a designated range of randomness. We predefined the number of classes to fit a GMM to our data. To determine an optimal number of classes automatically, we used Akaike’s Information Criterion (AIC) fit statistic to choose the best fitting GMM over varying numbers of components from one to five. We found that the AIC was minimized for the three-component GMM, in accordance with our manual categorization.

### Application to whole-slide images and histomorphological-genetic integration

To further investigate the robustness and versatility of ISHProfiler, we applied our workflow to an additional dataset, in which a whole tissue slide (108,000 × 138,000 pixels) of a transurethrally resected CRPC was hybridized with *PTEN* DISH and digitized with a Hamamatsu scanner. Using the same computational parameters and SVM classification model, our workflow classified more than one million *PTEN* and CEP10 signals (Fig. 3b–d), attaining considerable agreement with a serial section that was immunohistochemically stained with anti-*PTEN* antibody (Supplementary Fig. S9). The high-throughput application of ISHProfiler analysis to the whole slide allowed for the generation of a three-dimensional graph with each bar representing the ratio of *PTEN* to CEP10 for each individual local tumour foci (Supplementary Fig. S10). The bar graph reveals the complex intra-tumour CNV landscape of the *PTEN* locus and highlights focal *PTEN* ITH.

**Figure 4:**
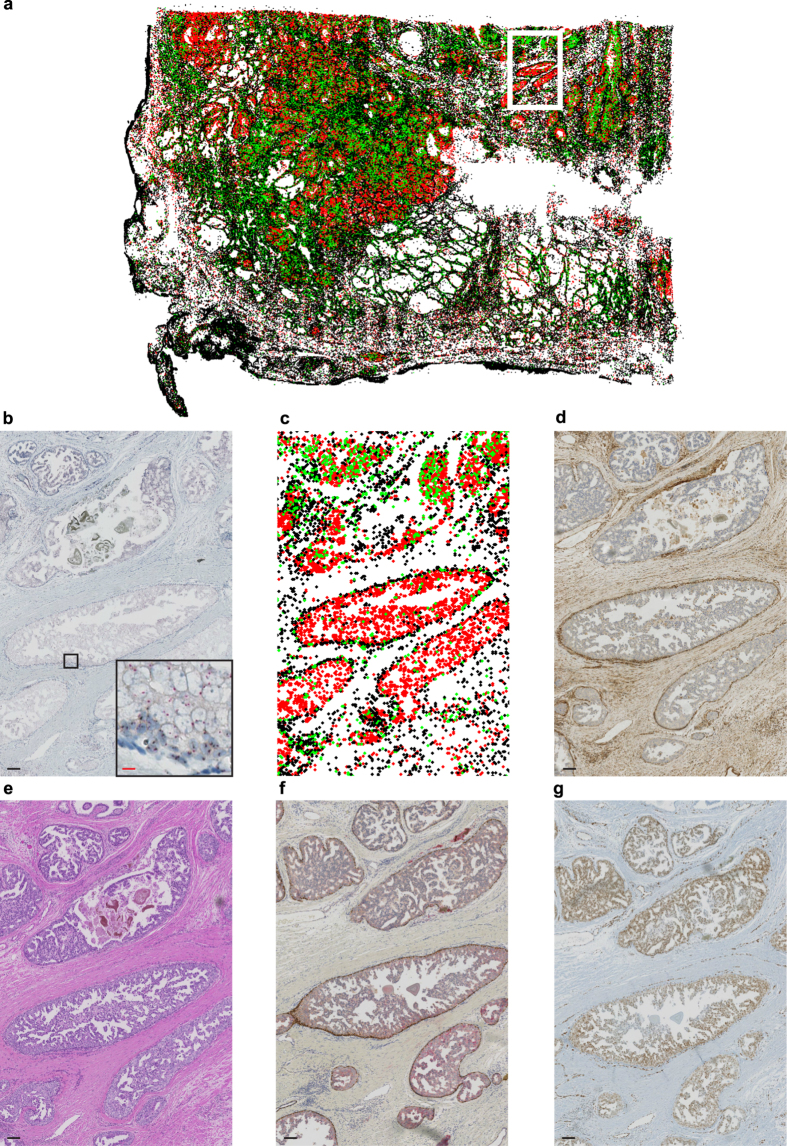
Analysis of IDC-P. (**a**) Whole slide image of IDC-P stained with *PTEN* DISH and visualized by a signal colour map as in Fig. 1g. (**b,c**) Zoomed versions of **A**, showing *PTEN* deletion in intra-ductal tumour cells in (**b)** and a signal colour map in **(c)**. Red scale bar, 10 μm and black scale bar, 100 μm. (**d**–**g**) Serial sections of the same tissue block. Scale bar 100 μm. PTEN IHC (**d**), hematoxylin-eosin (H&E) (**e**), PIN4 IHC (**f**), and ERG IHC (**g**).

Intra-ductal carcinoma of the prostate (IDC-P) represents an aggressive disease. PTEN loss has been reported as a potentially useful marker to distinguish IDC-P from high-grade prostatic intraepithelial neoplasia (PIN) by immunostaining in biopsy specimens with significant clinical implications^23^. On the basis of this observation, we stained a large tissue section of an IDC-P with *PTEN* DISH and visualized CNV using the signal colour map. The obtained heterogeneous topological pattern of molecular signals depicted *PTEN* homozygous deletion in the IDC-P component, in contrast to the retained *PTEN* status in the surrounding benign basal cells. This pattern was recapitulated in intra-ductal areas on serial sections stained with immunohistochemistry (IHC) (Fig. 4). This integrative analysis of genetic profiling with tissue morphology suggested that visualization by ISHPropfiler provides high-resolution single locus copy-number information in histomorphologically intact tissues, enabling the evaluation of ITH at single-cell level without compromising the ability to determine morphological and topological relationships of lesions.

### Generalizability tested for other genes and cancers

**Figure 5:**
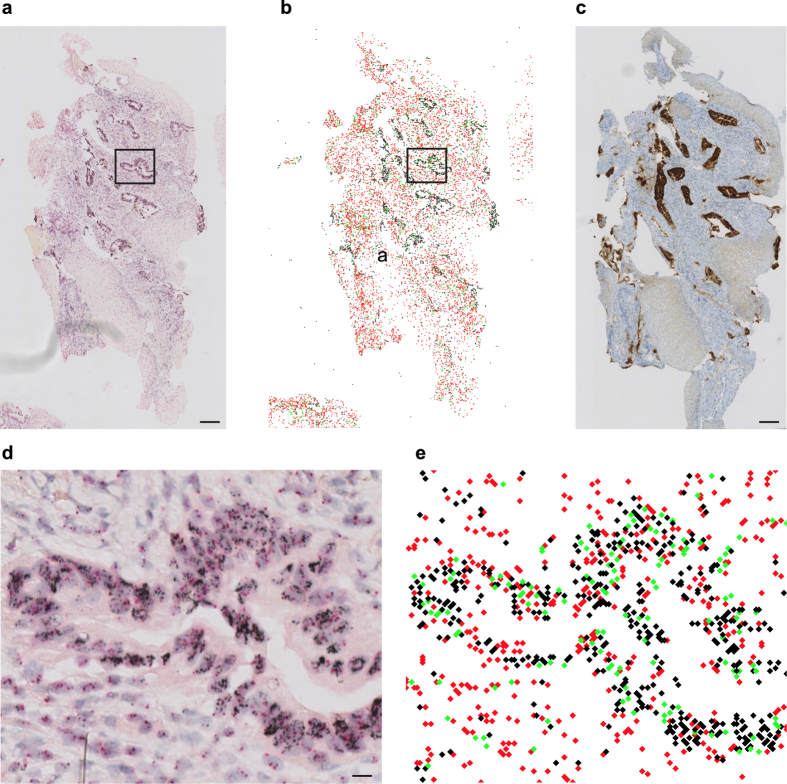
Analysis of HER2 in a whole slide tissue of gastric caner. (**a**) A selected area of a whole tissue slide of gastric cancer. Scale bar 100 μm. (**b**) A signal colour map with *HER2*, CEP17 and *HER2*+CEP17 illustrated by black, red, and green signals respectively. (**c**) A serial section immunohistochemically stained by anti-HER2 antibody. Scale bar 100 μm. (**d**) A zoomed image of (**a)**. Scale bar 10 μm. (**e**) A zoomed version of (**b)**.

Finally, we explored the general applicability of our ISHProfiler workflow by applying it to additional cancer entities and genetic loci. For measuring amplification of 19q12 including *CCNE1* and *URI* in ovarian^24^ and endometrial cancers, we used ISHProfiler to generate signal colour maps of two selected tissue cores (Supplementary Fig. S11). The global ratio of 19q12 to CEP19 and RLR at a threshold of 2.0 for both cancers closely matched manual assessment^24^. Moreover, we applied ISHProfiler to a whole slide of ovarian cancer and generated a signal colour map and a bar graph (Supplementary Fig. S12). Both figures showed no ITH as validated by manual inspection, demonstrating ISHProfiler’s capability to discriminate between ITH and homogenous events, even for different genes and cancer tissues. Furthermore, our ISHProfiler workflow successfully detected a heterogeneous *HER2* gene amplification in a well-differentiated gastric adenocarcinoma, hybridized with *HER2 and* CEP17 molecular probes. The amplified regions agreed with the overexpression of HER2 detected by immunohistochemical staining (Fig. 5).

## Discussion

**Figure 6:**
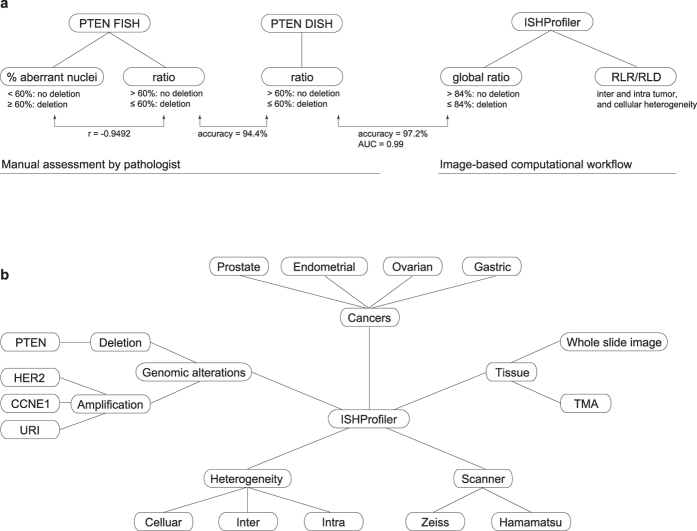
Workflow and applications of ISHProfiler. (**a**) A synopsis of the manual analysis of FISH and DISH, and the computational workflow ISHProfiler. (**b**) Applications of ISHProfiler.

Genetic ITH has critical impact on cancer diagnosis and treatment, and is associated with cancer evolution. Methods such as genome sequencing only provide single-cell information without spatial context, whereas stand-alone FISH and IHC can only evaluate ITH in a qualitative and subjective fashion. These obstacles have prevented ITH to be properly evaluated in research and clinical practice, leading to poor understanding of cancer evolution at single cell level. Our image-based computational workflow: ISHProfiler (Fig. 6 and Supplementary Methods) enabled for the first time the unbiased and reproducible quantification of genetic ITH on human cancer tissues with visualization tools, while simultaneously preserving the spatial and morphological information at single cell level. Therefore, clinical integration of our computational ISHProfiler bridges the gap between traditional molecular pathology and genomic studies.

The distribution of individual tissue spots with different degrees of CNV heterogeneity in the scatterplot (Fig. 3a) indicated that current grouping of tumor malignancy into discrete categories was subjective and arbitrary. Our integrative system with both cellular spatial organization and genetic ITH provides totally different information that is complementary to each other, essentially facilitating the investigation of patient outcome and the inference of tumor evolution.

Computation in ISHProfiler is high-throughput, because it neither relies on morphological features of cells, nor their detection, completely omitting computationally expensive algorithms such as segmentation, feature extraction and complex predictive modelling that are regarded as essential algorithmic steps for various image-based biological and translational studies^11^,^13^,^14^,^16^,^25^,^26^,^27^,^28^,^29^,^30^.

Manual analysis of genetic alterations based on molecular ISH signals relies solely on the expertise of trained pathologists. Visual inspection of histological slides and manual scoring of CNV are prone to inconsistent assessment among different pathologists, with different laboratory settings, and over extended project durations^10^,^11^,^12^. Besides, any tissue sample is a two-dimensional section from a three-dimensional specimen, thus the amount of genes and corresponding CEPs in each single cell may not reflect its bona-fide quantity. Notably, statistical averaging over multiple single-cell based ratio counting^9^,^12^,^24^,^31^ propagates the error at the single-cell level to a given tumour area, leading to inaccurate measurement of underlying genetic variations. Cellular and intra-tumour heterogeneity, intrinsic staining artefacts, and batch effects further complicate the assessment, especially when single-valued thresholds are used for dichotomizing genetic status. ISHProfiler alleviates these problems by using the region-based global ratio in conjunction with automatic thresholding by ROC analysis. Possible false positively or negatively detected or classified signals by ISHProfiler at single-cell resolution are irrelevant for the final dichotomization of genetic alteration (deletion, normal, or amplification) at the tissue core or whole slide resolution, because systematic and random errors are likely cancelled out when thousand and millions of molecular signals are involved in the calculation, while inconsistencies and biases associated with manual assessment is more error-prone^10^,^11^,^12^. Additionally, RLR and RLD take advantage of local distribution in random neighbourhoods, which allows us to quantify tumour heterogeneity as distribution.

Our ISHProfiler was able to detect allelic gains and loss in different tumor tissues, independent of genes of interest and associated CEPs, without any parameter adjustment for point detection and classification, if similar DISH staining protocols and digitization procedures were used. However, some of the limitations are the need for parameter tuning of the circular Hough transform and the construction of a new training image set for re-validating the classification model, if the DISH staining or image acquisition is performed in another laboratory under different conditions. However, this recalibration problem can be solved by implementing highly sensitive point detection algorithms, building a graphic user interface for efficient expert annotation of multiple genes, and providing a set of classification models for distinct DISH assays, staining protocols, and digitization procedures.

Genomics and proteomics studies rely on the selection of patient tissues by trained pathologists^32^,^33^,^34^. Such selection has traditionally been accomplished by staining whole slide tissues with H&E, IHC or ISH, followed by manual evaluation of small selected regions of interest. Our quantitative signal colour map, which preserves tissue topology and combines genetic analysis with clinico-pathological assessment, will provide accurate and objective punch guidance for the optimal hotspot selection of heterogeneous tumour tissues.

Our generic ISHProfiler can be potentially used for reliable quantification of heterogeneous allelic gains and losses of any gene in any tissue specimen hybridized at single-cell level, thereby enabling precise patient stratification and permitting broad applications in tissue-based biomedical research.

## Methods

### Study design and reporting

We have used a *PTEN* FISH PC cohort, a *PTEN* DISH PC dataset, several DISH whole slide images of *PTEN*, 19q12, and HER2, and two tissue cores stained with DISH probes for 19q12. In our retrospective PC cohort, *PTEN* deletion expression could be observed in 78 of 339 (23%) RPE specimens. We further estimated that the occurrence of *PTEN* deletion expression would double the risk of death during follow up, resulting in a hazard ratio of 2.0. The estimation of statistical power versus total sample size *N* for different hazard ratios was shown in (Supplementary Fig. S13). Accordingly, the available sample size of 339 analysable patients would be sufficient to detect a difference concerning death recurrence with a significance of *P* < 0.05 and a power of almost 100%. For transparent and complete reporting of the prognostic role of *PTEN* FISH we followed the REporting recommendations for tumour MARKer prognostic studies (REMARK)^32^.

### Cancer patient samples

A total of 424 FFPE tissue samples were retrieved from the archives of the Institute of Surgical Pathology, University Hospital Zurich, Switzerland^32^,^35^,^36^,^37^,^38^. H&E-stained slides of all specimens were evaluated by two experienced pathologists to identify representative areas for tissue microarray (TMA) construction. One tissue core (diameter 0.6 mm and thickness of 4 μm) of a representative tumour area per patient was taken from a “donor” block and arranged in a new “recipient” block using a customized instrument. Tumour stage and Gleason score of the cohort were assigned according to the International Union Against Cancer (UICC) and WHO/ISUP criteria. The study was approved by the Cantonal Ethics Committee of Zurich (StV-No. 2008-0025) and the associated methods were carried out in accordance with the approved guidelines. Effectively, 424 samples were used in the FISH analysis and a subset of 71 samples was used for DISH manual assessment and computational analysis.

Detection of the 19q12 amplicon using the recently established DISH assay was performed as previously described for the ovarian and endometrial cancers^24^,^39^. The amplification status of the 19q12 region was identified using a DNA probe set (Ventana) by measuring the copy number ratio of the 19q12 amplicon (black signals) to the CEP19 (red signals). The 19q12 DISH probe is a DNA probe that covers approximately 560 kb containing the coding sequences of *CCNE1* and *URI.* Silver-enhanced, bright-field *HER2* (black signals) and CEP17 (red signals) DISH staining in the gastric cancer^40^ was performed using the respective kits (Ventana) according to published procedures^31^,^40^. Analysis of ovarian, endometrial, and gastric cancer patients was approved by the Cantonal Ethics Committee of Zurich (KEK-ZH-No. 2014-0604) and the associated methods were carried out in accordance with the approved guidelines.

### *PTEN* FISH analysis

For *PTEN* deletion analysis, a dual-colour FISH was performed using commercially available DNA probes for the region 10q23.3 (Spectrum Orange, *PTEN* locus-specific probe; Abbott Molecular) and 10p11.1-q11.1 (Spectrum Green, centromeric probe (CEP) of chromosome 10; LSI *PTEN*/CEP10; Abbott Molecular), as described previously^12^. Each tissue core was evaluated for each FISH probe by manually counting signals in 20–60 intact non-overlapping interphase nuclei, using a fluorescence microscope (Leica DM6000 B). In case of insufficient staining (n = 5), additional sections of the FFPE tissue blocks were hybridized with *PTEN* FISH. Manual scoring was performed in tumour areas with loss of *PTEN* signals. The average of two experienced pathologists manual, independent assessment led to the final score. Two scoring methods were used: the percentage of aberrant nuclei and the ratio of *PTEN* to CEP10 signals. As threshold for *PTEN* deletion, the percentage of aberrant nuclei was used in accordance to previous publications^9^: hemizygous *PTEN* deletion was defined as the presence of fewer *PTEN* signals than CEP10 signals in at least 60% of counted nuclei. Homozygous *PTEN* deletion was defined if at least one third (33%) of aberrant nuclei revealed zero *PTEN* signal in a tissue core, with the presence of one or two *PTEN* signals in adjacent normal cells. Accordingly, *PTEN* deletion was defined if the average ratio of *PTEN* to CEP10 signals was less than or equal to 60%.

### *PTEN* DISH analysis

A BenchMark ULTRA automated stainer was used for the optimization and performance evaluation of the DISH assay for CEP10 and *PTEN* DNA targets. In this assay, a black signal represents the *PTEN* probe a red signal corresponds to the CEP10, which were visualized with *ultraView* SISH DNP and Red ISH DIG detection kit respectively, after hybridization with the *PTEN* DNP probe and CEP10 probe cocktail. All tissue sections were counterstained with hematoxylin II and bluing reagent (Ventana). The threshold of 60% for the ratio was used.

### *ALU II* silver *in situ* hybridization (SISH)

Another gene probe, *ALU II*, an important group of widely distributed sequence repeats in the human genome, was used as a positive control for viable DNA on the same tissue specimens and detected by a single colour SISH assay. For *ALU II* SISH, the *ALU* gene target was visualized with *ultraView* SISH DNP detection kit after hybridization with the *ALU II* DNP Probe (Ventana). A total of 13 cores were excluded from further analyses because of unviable DNA (Supplementary Fig. S4), lack of target tissue, or weak CEPs.

### *ERG* break-apart FISH and SPOP mutation

We used a FISH assay to detect *ERG* rearrangement at the chromosomal level on FFPE specimens. Hence, we performed a split-signal-approach, with two probes spanning the ERG locus as described earlier^41^. Two experienced pathologists independently assessed all cases and at least 100 nuclei per case were evaluated. The assay is also capable of differentiating between two different mechanisms of *ERG* rearrangement. Methods and results of *SPOP* mutation analysis have already been published in part previously^36^.

### Immunohistochemistry (IHC)

IHC was performed using a Ventana Benchmark automated staining system with two-micrometer TMA tissue sections. Two pathologists performed a blinded evaluation of the immunostained slides without knowledge of clinical data. Cytoplasmic PTEN and nuclear immunoreactivity of ERG was estimated using a semi-quantitative four-step scoring system (0–3): 0, negative; 1, weak positive; 2, strong positive; 3, very strong positive. For negative controls, the primary antibody was omitted. The specificity of the ERG antibody has been thoroughly validated in former studies^42^. The following antibodies were used for IHC: anti-ERG (Ventana; EPR3864), anti-PTEN (Dako; clone 6H2.1), anti-HER2 (Ventana; PATHWAY HER2, clone 4B5). In addition, an antibody cocktail comprising p63, CK5, CK14, and P504S (Biocare Medical; PIN-4 Cocktail) was used.

### Statistical analysis

Statistical association between clinic-pathological and molecular parameters was tested by two-sided Fisher’s exact test or Pearson’s chi-squared test. Nonparametric Kaplan-Meier estimators were used to analyze overall, disease-specific and recurrence-free survival. Patients were censored at the time of their last clinical follow-up visit. Simultaneous 95% confidence bands were computed for the whole range of time values. Differences between survival estimates were evaluated by the log-rank test. The threshold for statistical significance was set to *P* < 0.05. Univariate and multivariable Cox regression models were estimated. Multivariate stepwise reverse selection was set to *P* = 0.1 as the limit. In the forest plot (Supplementary Fig. S3), the dashed line was drawn at the no effect point (hazard ratio of 1.0). Horizontal lines represent a 95% CI. The mid-point of the box denotes the mean effect estimate and the area of the box represents the weight for each subgroup. Statistical analyses were performed using PASS (2008), survival, OIsurv, and metaphor packages in R (version 3.2.0), and SPSS (version 22.0).

In the ROC analysis, we used global ratios as the classifier prediction scores and DISH manual assessment as true class labels. Point wise confidence bands for the area under the curve were computed by generating 1,000 bootstrap replicates. The optimal operating point by the false positive rate and true positive rate was obtained by finding the slope that satisfies the optimality criterion. The optimal operating point determined the optimal threshold, which was then used to dichotomize the global ratios for determining genetic status.

We assume the random variables (RLR and RLD) are independent and identically distributed and the constructed features for the GMM are normally distributed.

### Image digitization

The bright-field and fluorescence slide scanner Axio Scan.Z1 (Carl Zeiss) was used to digitize tissue cores with a resolution of x40 (0.11 μm/pixel) according to manufacturer’s instructions. To show the general applicability of our ISHProfiler, we also used a C9600 NanoZoomer 2.0-HT Digital slide scanner from (Hamamatsu) with a resolution of 40x (0.23 μm/pixel). The Zeiss scanner was used for digitizing all prostate cancer tissue cores and an ovarian cancer tissue core, and the Hamamatsu scanner for digitizing an endometrial cancer tissue core, and whole slides of prostate, ovarian and gastric cancers.

### Image-based computational workflow (ISHProfiler)

Tissue cores or slides were digitized and pre-processed (white balancing, deconvolution, and contrast modification) using the scanner’s default auto-correction settings. Images were then resized by bicubic interpolation to 4096 × 4096 pixels for efficient tiling (4096 = 2^12^) and served as input data for the computational workflow ISHProfiler. Pseudocode of the ISHProfiler, details about the parameter tuning, and applications of ISHProfiler to other genes are provided in the Supplementary Information.

The RLR and RLD were calculated as follows. First, a predefined number of random points were generated, such that these points were uniformly distributed over an entire tissue core, yet as few gene and CEP signals as possible were sampled without replacement (default set to 300, Supplementary Fig. S8). The coordinate of each random point was then substituted by the coordinate of its closest CEP point. Second, in the neighborhood of such a CEP point, all gene and CEP points that lay within a predefined radius (default set to 60 pixels, such that at least one adjacent cell was included in the neighborhood) were recorded. Third, for each neighborhood, the ratio was the division of all genes by all CEP signals and the density was the total number of gene and CEP signals. The ratio and density distributions over the neighborhoods were defined as the RLR and RLD respectively.

For each tissue core, we extracted mean, median and standard error of the mean from the distributions of the RLR and RLD (3 × 2 = 6 dimensions), to which we performed principal component analysis (PCA) for reducing the dimensionality from six to two. In the two dimensional PCA subspace, we then applied a probabilistic model by Gaussian mixture modeling (GMM) to cluster the 71 cores into three classes. By incorporating the domain knowledge that homogeneous deletion exhibits a lower gene to CEP ratio than cases with homogeneous non-deletion and multi-level heterogeneity, we could locate the centroid of this homogeneous deletion class (centroid C1 in Fig. 3A) and calculated the Mahalanobis distances of each point to it.

The computational workflow was implemented in MATLAB (R2014b) and tested on a MacPro (2014). MATLAB built-in functions for the circular Hough transform (imfindcircles) and ROC analysis (perfcurve) were used. The software package LIBSVM^43^ (version 3.18) was used to train, validate and test SVM models on the data.

### Data and materials availability

The source code for the method presented in this manuscript is provided in the Supplementary Software and on www.wildlab.ch/ish. The dataset of 71 tissue core images is available in the online repository Harvard Dataverse: https://dataverse.harvard.edu/dataset.xhtml?persistentId=doi:10.7910/DVN/RRKMHC. In addition, the MATLAB code with basic ISHProfiler functionalities has been deployed as a Java plugin for the free open source software TMARKER (http://www.nexus.ethz.ch/equipment_tools/software/tmarker.html).

## Additional Information

**How to cite this article**: Zhong, Q. *et al*. Image-based computational quantification and visualization of genetic alterations and tumour heterogeneity. *Sci. Rep.* **6**, 24146; doi: 10.1038/srep24146 (2016).

## Supplementary Material

Supplementary Information

## References

[b1] Swanton C. Intratumor heterogeneity: evolution through space and time. Cancer Res. 72, 4875–4882 (2012). 23002210 10.1158/0008-5472.CAN-12-2217 PMC3712191

[b2] Marusyk A., Almendro V. & Polyak K. Intra-tumour heterogeneity: a looking glass for cancer? Nat. Rev. Cancer 12, 323–334 (2012). 22513401 10.1038/nrc3261

[b3] Longo D. L. Tumor heterogeneity and personalized medicine. N. Engl. J. Med. 366, 956–957 (2012). 22397658 10.1056/NEJMe1200656

[b4] Cancer Genome Atlas Research, N. . The Cancer Genome Atlas Pan-Cancer analysis project. Nat. Genet. 45, 1113–1120 (2013). 24071849 10.1038/ng.2764 PMC3919969

[b5] Janiszewska M. . *In situ* single-cell analysis identifies heterogeneity for PIK3CA mutation and HER2 amplification in HER2-positive breast cancer. Nat. Genet. 47, 1212–1219 (2015). 26301495 10.1038/ng.3391 PMC4589505

[b6] Wolff A. C. . American Society of Clinical Oncology/College of American Pathologists guideline recommendations for human epidermal growth factor receptor 2 testing in breast cancer. Arch. Pathol. Lab. Med. 131, 18–43 (2007). 19548375 10.5858/2007-131-18-ASOCCO

[b7] Reid A. H. . Significant and sustained antitumor activity in post-docetaxel, castration-resistant prostate cancer with the CYP17 inhibitor abiraterone acetate. J. Clin. Oncol. 28, 1489–1495 (2010). 20159823 10.1200/JCO.2009.24.6819 PMC2849770

[b8] Reid A. H. . Novel, gross chromosomal alterations involving PTEN cooperate with allelic loss in prostate cancer. Mod. Pathol. 25, 902–910 (2012). 22460813 10.1038/modpathol.2011.207

[b9] Krohn A. . Genomic deletion of PTEN is associated with tumor progression and early PSA recurrence in ERG fusion-positive and fusion-negative prostate cancer. Am. J. Pathol. 181, 401–412 (2012). 22705054 10.1016/j.ajpath.2012.04.026

[b10] Fuchs T. J. & Buhmann J. M. Computational pathology: challenges and promises for tissue analysis. Comput. Med. Imag. Grap. 35, 515–530 (2011). 10.1016/j.compmedimag.2011.02.006 21481567

[b11] Zhong Q., Busetto A. G., Fededa J. P., Buhmann J. M. & Gerlich D. W. Unsupervised modeling of cell morphology dynamics for time-lapse microscopy. Nat. Methods 9, 711–713 (2012). 22635062 10.1038/nmeth.2046

[b12] Cima I. . Cancer genetics-guided discovery of serum biomarker signatures for diagnosis and prognosis of prostate cancer. Proc. Natl. Acad. Sci. USA 108, 3342–3347 (2011). 21300890 10.1073/pnas.1013699108 PMC3044355

[b13] Beck A. H. . Systematic analysis of breast cancer morphology uncovers stromal features associated with survival. Sci. Transl. Med. 3, 108ra113 (2011). 10.1126/scitranslmed.3002564 22072638

[b14] Yuan Y. . Quantitative image analysis of cellular heterogeneity in breast tumors complements genomic profiling. Sci. Transl. Med. 4, 157ra143 (2012). 10.1126/scitranslmed.3004330 23100629

[b15] Wilkens L. . Detection of chromosomal aberrations in well-differentiated hepatocellular carcinoma by bright-field *in situ* hybridization. Mod. Pathol. 15, 470–475 (2002). 11950923 10.1038/modpathol.3880548

[b16] Schuffler P. J. . TMARKER: A free software toolkit for histopathological cell counting and staining estimation. J. Pathol. Inform. 4, S2 (2013). 23766938 10.4103/2153-3539.109804 PMC3678753

[b17] Duda R. O. & Hart P. E. Use of the Hough transformation to detect lines and curves in pictures. Commun. ACM 15, 11–15 (1972).

[b18] Cortes C. & Vapnik V. Support-Vector Networks. Mach. Learn. 20, 273–297 (1995).

[b19] Krohn A. . Heterogeneity and chronology of PTEN deletion and ERG fusion in prostate cancer. Mod. Pathol. 27, 1612–1620 (2014). 24762546 10.1038/modpathol.2014.70

[b20] Hollander M. C., Blumenthal G. M. & Dennis P. A. PTEN loss in the continuum of common cancers, rare syndromes and mouse models. Nat. Rev. Cancer 11, 289–301(2011). 21430697 10.1038/nrc3037 PMC6946181

[b21] Haffner M. C. . Tracking the clonal origin of lethal prostate cancer. J. Clin. Invest. 123, 4918–4922 (2013). 24135135 10.1172/JCI70354 PMC3809798

[b22] Carver B. S. . Aberrant ERG expression cooperates with loss of PTEN to promote cancer progression in the prostate. Nat. Genet. 41, 619–624 (2009). 19396168 10.1038/ng.370 PMC2835150

[b23] Lotan T. L. . Cytoplasmic PTEN protein loss distinguishes intraductal carcinoma of the prostate from high-grade prostatic intraepithelial neoplasia. Mod. Pathol. 26, 587–603 (2013). 23222491 10.1038/modpathol.2012.201 PMC3610824

[b24] Noske A. . Characterization of the 19q12 amplification including CCNE1 and URI in different epithelial ovarian cancer subtypes. Exp. Mol. Pathol. 98, 47–54 (2015). 25527175 10.1016/j.yexmp.2014.12.004

[b25] Carpenter A. E. . CellProfiler: image analysis software for identifying and quantifying cell phenotypes. Genome Biol. 7, R100 (2006). 17076895 10.1186/gb-2006-7-10-r100 PMC1794559

[b26] Swedlow J. R., Goldberg I. G. & Eliceiri K. W. & Consortium, O. M. E. Bioimage informatics for experimental biology. Annu. Rev. Biophys. 38, 327–346 (2009). 19416072 10.1146/annurev.biophys.050708.133641 PMC3522875

[b27] Held M. . CellCognition: time-resolved phenotype annotation in high-throughput live cell imaging. Nat. Methods 7, 747–754 (2010). 20693996 10.1038/nmeth.1486

[b28] Peng H., Bateman A., Valencia A. & Wren J. D. Bioimage informatics: a new category in Bioinformatics. Bioinformatics 28, 1057 (2012). 22399678 10.1093/bioinformatics/bts111 PMC3324521

[b29] Neumann B. . Phenotypic profiling of the human genome by time-lapse microscopy reveals cell division genes. Nature 464, 721–727 (2010). 20360735 10.1038/nature08869 PMC3108885

[b30] Jaqaman K. . Robust single-particle tracking in live-cell time-lapse sequences. Nat. Methods 5, 695–702 (2008). 18641657 10.1038/nmeth.1237 PMC2747604

[b31] Stenzinger A. . Quantitative analysis of diagnostic guidelines for HER2-status assessment. J. Mol. Diagn. 14, 199–205 (2012). 22500949 10.1016/j.jmoldx.2012.01.012

[b32] Barbieri C. E. . Exome sequencing identifies recurrent SPOP, FOXA1 and MED12 mutations in prostate cancer. Nat. Genet. 44, 685–689 (2012). 22610119 10.1038/ng.2279 PMC3673022

[b33] Guo T. . Rapid mass spectrometric conversion of tissue biopsy samples into permanent quantitative digital proteome maps. Nat. Med. 21, 407–413 (2015). 25730263 10.1038/nm.3807 PMC4390165

[b34] Liu Y. . Glycoproteomic analysis of prostate cancer tissues by SWATH mass spectrometry discovers N-acylethanolamine acid amidase and protein tyrosine kinase 7 as signatures for tumor aggressiveness. Mol. Cell. Proteomics 13, 1753–1768 (2014). 24741114 10.1074/mcp.M114.038273 PMC4083113

[b35] Mortezavi A. . KPNA2 expression is an independent adverse predictor of biochemical recurrence after radical prostatectomy. Clin. Cancer Res. 17, 1111–1121 (2011). 21220479 10.1158/1078-0432.CCR-10-0081

[b36] Blattner M. . SPOP mutations in prostate cancer across demographically diverse patient cohorts. Neoplasia 16, 14–20 (2014). 24563616 10.1593/neo.131704 PMC3924544

[b37] Haldrup C. . DNA methylation signatures for prediction of biochemical recurrence after radical prostatectomy of clinically localized prostate cancer. J. Clin. Oncol. 31, 3250–3258 (2013). 23918943 10.1200/JCO.2012.47.1847

[b38] Kristensen H. . Hypermethylation of the GABRE ~ miR-452 ~ miR-224 promoter in prostate cancer predicts biochemical recurrence after radical prostatectomy. Clin. Cancer Res. 20, 2169–2181 (2014). 24737792 10.1158/1078-0432.CCR-13-2642

[b39] Ikenberg K. . KPNA2 is overexpressed in human and mouse endometrial cancers and promotes cellular proliferation. J. Pathol. 234, 239–252 (2014). 24930886 10.1002/path.4390

[b40] Ruschoff J. . HER2 testing in gastric cancer: a practical approach. Mod. Pathol. 25, 637–650 (2012). 22222640 10.1038/modpathol.2011.198

[b41] Tomlins S. A. . Recurrent fusion of TMPRSS2 and ETS transcription factor genes in prostate cancer. Science 310, 644–648 (2005). 16254181 10.1126/science.1117679

[b42] Park K. . Antibody-based detection of ERG rearrangement-positive prostate cancer. Neoplasia 12, 590–598 (2010). 20651988 10.1593/neo.10726 PMC2907585

[b43] Chang C.-C. & Lin C.-J. LIBSVM: A library for support vector machines. ACM Trans. Intell. Syst. Technol. 2, 1–27 (2011).

