# Supplementary material for: Image-based computational quantification and visualization of genetic alterations and tumour heterogeneity
Source: Sci Rep. 2016 Apr 7;6:24146. doi: 10.1038/srep24146 (PMC4823793; doi:10.1038/srep24146)
Supplement: Supplementary Information [file srep24146-s1.pdf]

# Supplementary Information

# Image-based computational quantification and visualization of genetic alterations and tumour heterogeneity

Qing Zhong[1#], Jan H Rüschoff[1#], Tiannan Guo[2], Maria Gabrani[3], Peter J Schüffler[4], Markus Rechsteiner[1], Yansheng Liu[2], Thomas J Fuchs[5], Niels J Rupp[1], Christian Fankhauser[1], Joachim M Buhmann[2], Sven Perner[6], Cédric Poyet[7], Miriam Blattner[8], Davide Soldini[1], Holger Moch[1], Mark A Rubin[8], Aurelia Noske[1], Josef Rüschoff[9], Michael C Haffner[10], Wolfram Jochum[11], Peter J Wild[1*]

[1]Institute of Surgical Pathology, University Hospital Zurich, Zurich, Switzerland.
[2]Institute of Molecular Systems Biology, ETH, Zurich, Switzerland.
[3]Zurich Labouratory, IBM Research-Zurich, Rueschlikon, Switzerland.
[4]Department of Computer Science, ETH, Zurich, Switzerland.
[5]Memorial Sloan Kettering Cancer Center, New York, NY, USA.
[6]Department of Prostate Cancer Research, Institute of Pathology, University Hospital of Bonn, Bonn, Germany.
[7]Department of Urology, University Hospital Zurich, Zurich, Switzerland.
[8]Institute for Precision Medicine and Department of Pathology and Laboratory Medicine, Weill Medical College of Cornell University and New York-Presbyterian Hospital, New York, NY, USA.
[9]Targos Molecular Pathology, Pathology Nordhessen, Kassel, Germany.
[10]Sidney Kimmel Comprehensive Cancer Center, Johns Hopkins University, Baltimore, Maryland, USA.
[11]Institute of Pathology, Cantonal Hospital St. Gallen, St. Gallen, Switzerland.

*To whom correspondence should be addressed: peter.wild@usz.ch.

[#]These authors contributed equally to this work.

**Table of Contents**

Supplementary Methods Page 1 – 2

Supplementary Figures 1 – 13 Page 3 – 15

Supplementary Tables 1 – 3 Page 16 – 18

# Supplementary Methods

For the circular Hough transform, the signal radius were defined empirically from 1 to 7 pixels according to domain knowledge and the edge gradient threshold was set to Matlab default (Otsu's method). The detection sensitivity was set to Matlab default (0.85) for tissues scanned by the Zeiss scanner and was set to 0.95 for tissues digitized by the Hamamatsu scanner, because the Zeiss scanner has a higher scanning resolution and a more advanced image sensor.

The SVM model was trained and validated (5-fold cross validation and grid search that iterates overall all pairs of $C$ and $\gamma$) on an independent image set from a single tissue spot with two sets of expert annotations (one for the HER2 and the other for the remaining genes), consisting of 1000 image patches of size $13 \times 13$ pixels with *PTEN*, CEP10, *PTEN*+CEP10, white (background noise) and blue (cell stains) signals in the center of the patch (fig. S6). The feature vector was constructed by concatenating ($13 \times 13 = 169$) RGB values. For reduction of misclassified signals, only gene and corresponding CEP signals were used for subsequent calculation. Signals classified as white or blue were discarded. The maximum of the global ratio was set to three to circumvent false positive gene signals due to unspecific staining (any roundish black signals) for cases with gene deletion.

For prostate and ovarian cancers, each whole slide image was tiled into sub-images, in which we used the same parameter settings as the TMA for the circular Hough transform and the SVM model to detect and classify gene and CEP signals. A signal colormap was then drawn for each sub-image. By merging signal colormaps of all sub-images, the complete signal colormap of the whole slide was generated. A three-dimensional bar graph was plotted for visualizing intra-tumor heterogeneity, where each bar represents a sub-image. The same workflow of the ISHProfiler was also applied to a whole slide of a gastric cancer tissue stained with DISH probes for *HER2*/CEP17. To re-calibrate the molecular signal intensities of *HER2*/CEP17, we used a different expert annotation of the same training data for SVM training and cross-validation.

**Algorithm:** An image-based computational workflow: ISHProfiler

**input** : A digital image: `I` (tissue core or whole slide),
Radius range: `radiusRange`,
Detection sensitivity: `sensitivity`,
Neighborhood distance: `radius`,
$K$ random points: $P := \{p_k\}_{k=1}^{K}$,
A trained and validated SVM.

**output**: The global ratio, randomized local ratio (RLR), randomized local density (RLD), and a signal colormap.

Detect GENE and CEP signals by circular Hough transform: `imfindcircles(I,radiusRange,sensitivity)`. Get positions of the detected signals.

Using the SVM to classify the detected signals into five classes and retain only three classes, GENE: $G := \{g_n\}_{n=1}^{N}$, CEP: $C := \{c_m\}_{m=1}^{M}$ and GENE+CEP: $Z := \{z_l\}_{l=1}^{L}$, where $g_n$ denotes a single occurrence of the gene.

**Return** the global ratio $= \dfrac{\sum_{n=1}^{N} g_n + \sum_{l=1}^{L} z_l}{\sum_{m=1}^{M} c_m + \sum_{l=1}^{L} z_l}$.

Set the maximum global ratio for filtering false positive cases (optional).

**Plot** a signal colormap according to signal positions and colors in $G, C$ and $M$.

**foreach** *Random points* $p_k$ **do**

- find the closest CEP point to $p_k$: $c_k^* = \arg\min_c ||c - p_k||$, where $|| \cdot ||$ denotes the Euclidean distance.
- find all GENE and CEP signals $S_k = \{G_k \vee C_k \vee Z_k\}$ in the neighborhood, such that $||S_k - c_k^*|| <$ `radius`.
- Calculate the ratio of GENE to CEP: $r_k = \frac{|G_k|+|Z_k|}{|C_k|+|Z_k|}$ in the neighborhood, where $| \cdot |$ denotes the cardinality of a set.
- Calculate the total number of GENE and CEP: $d_k = |G_k| + |C_k| + |Z_k|$ in the neighborhood.

**end**

Save all $r_k, d_k, k = 1, 2..., K$.

**Return** the $RLR := \{r_k\}_{k=1}^{K}$ and $RLD := \{d_k\}_{k=1}^{K}$ as distributions, $\frac{1}{K}\sum_{k=1}^{K} r_k$ and $\frac{1}{K}\sum_{k=1}^{K} d_k$ as means, respectively.

**Return** a feature vector $\mathbf{x} = (x^1, x^2, ..., x^6) \in \mathbb{R}^6$ with six dimensions, representing the image `I`. The features $x^1, x^2, x^3$ are the mean, median and the s.e.m. of the RLR, and $x^4, x^5, x^6$ are the mean, median and the s.e.m. of the RLD.

# Supplementary Figures

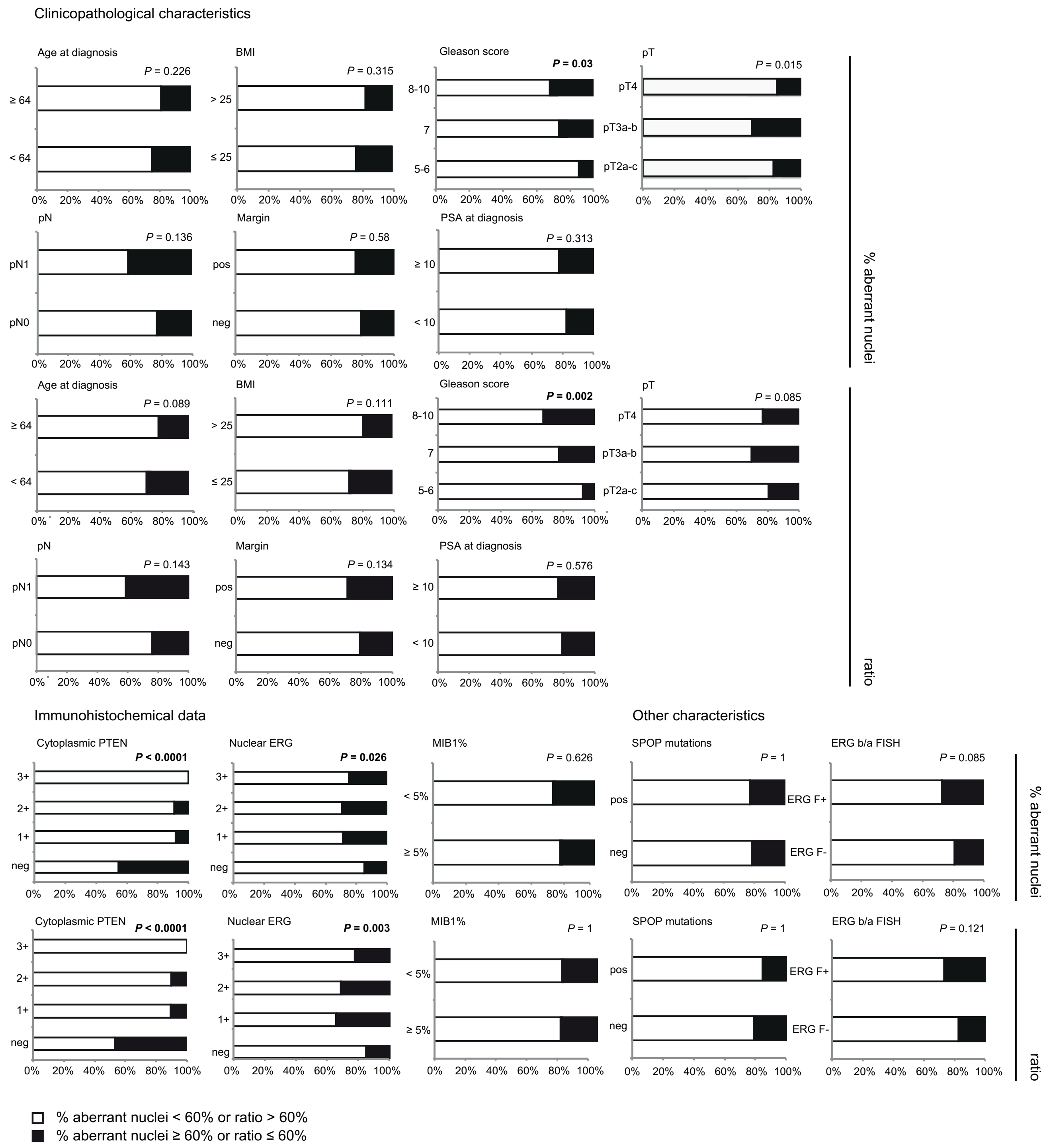


**Supplementary Figure S1.** Comparison between *PTEN* FISH percent aberrant nuclei and ratio for various features. Two-sided Fisher's exact test or Pearson's chi-squared test were used. $P < 0.05$ are marked in bold.

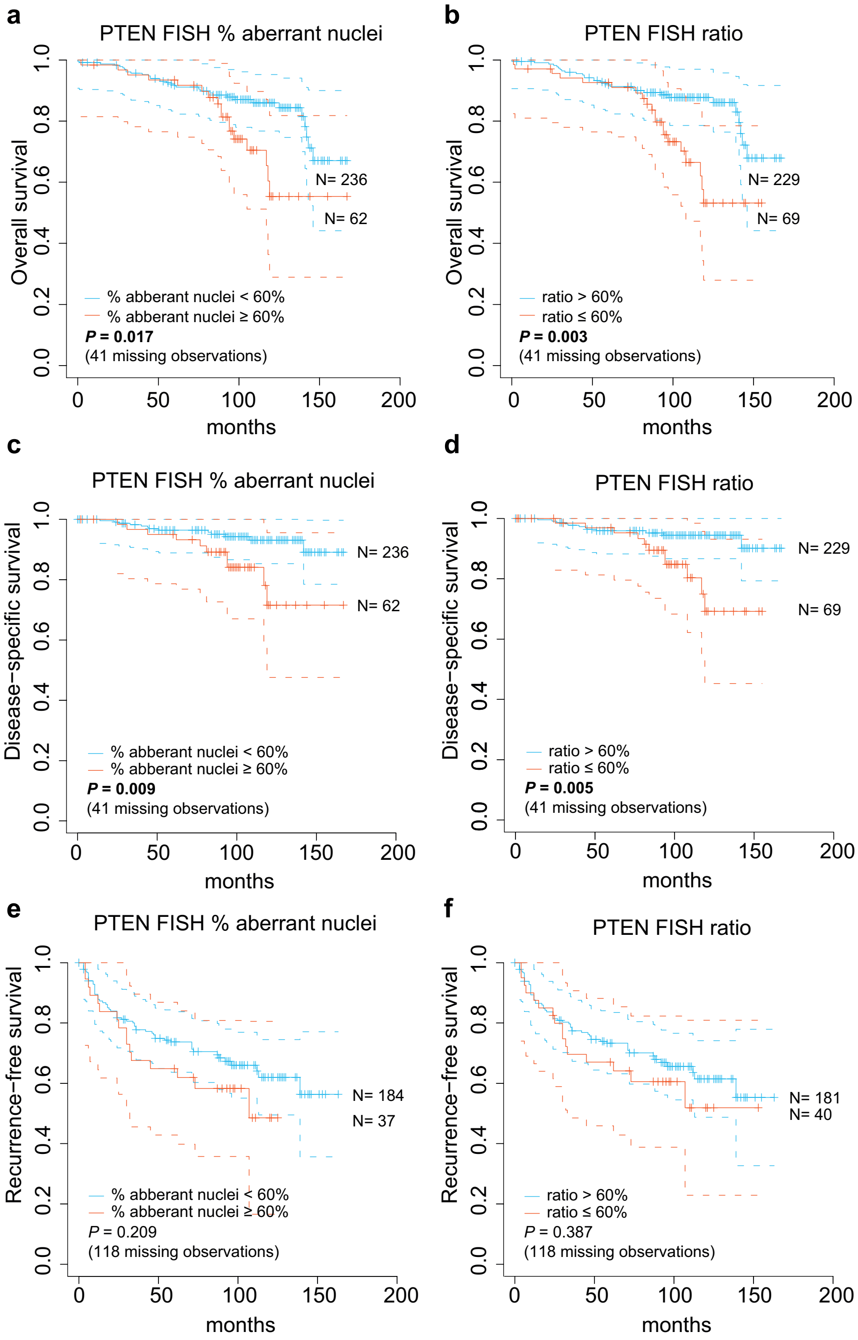


**Supplementary Figure S2.** Identification of patient outcome by *PTEN* FISH percentage aberrant nuclei and ratio. (**a**) Kaplan-Meier curves with simultaneous 95% confidence bands of patient overall survival over time after diagnosis when patients were dichotomized into two groups by the percentage aberrant nuclei. Vertical lines illustrate censored patients. Log-rank tests were performed to test for equality in the survival expectation in each group. $N$ values represent the number of patients in each group under risk. $P < 0.05$ are marked in bold. (**b**) Overall survival by the ratio. (**c**) Disease-specific survival by the percentage aberrant nuclei. (**d**) Disease-specific survival by the ratio. (**e**) Recurrence-free survival by the percentage aberrant nuclei (**f**) Recurrence-free survival by the ratio.

| Variable | Characteristics | Overall survival (hazard ratio [95% confidence interval]) | *P* value |
|---|---|---|---|
| **UNIVARIATE COX REGRESSION** | | | |
| Age at diagnosis | < 64 y, ≥ 64 y | 1.29 [ 0.73, 2.30 ] | = 0.380 |
| BMI | ≤ 25, > 25 (kg/m$^2$) | 1.02 [ 0.53, 1.95 ] | = 0.961 |
| Gleason score | 5-6, 7, 8-10 | 2.38 [ 1.48, 3.85 ] | **< 0.001** |
| pT | pT2a-c, pT3a-b, pT4 | 2.06 [ 1.33, 3.20 ] | **= 0.001** |
| pN | pN0, pN1 | 1.90 [ 0.75, 4.80 ] | = 0.176 |
| Margin status | no tumor, positive surgical margin | 1.96 [ 1.11, 3.46 ] | **= 0.020** |
| PSA at diagnosis | <10, ≥ 10 (ng/µl) | 0.91 [ 0.50, 1.66 ] | = 0.753 |
| PTEN FISH ratio | no deletion (> 60%), deletion (≤ 60%) | 2.31 [ 1.31, 4.09 ] | **= 0.004** |
| SPOP mutation | wide type, mutation | 0.56 [ 0.07, 4.14 ] | = 0.568 |
| ERG b/a FISH | no/with rearrangement | 0.93 [ 0.50, 1.74 ] | = 0.825 |
| PTEN IHC | negative, score 1, 2, 3 | 0.74 [ 0.52 ,1.07 ] | = 0.115 |
| **MULTIVARIATE COX REGRESSION** | | | |
| **Global model** | | | |
| Gleason score | 5-6, 7, 8-10 | 2.02 [ 1.17 , 3.47 ] | = **0.0113** |
| pT | pT2a-c, pT3a-b, pT4 | 1.30 [ 0.76 , 2.22 ] | = 0.3472 |
| Margin status | no tumor, positive surgical margin | 1.49 [ 0.78 , 2.82 ] | = 0.2239 |
| PTEN FISH ratio | no deletion (> 60%), deletion (≤ 60%) | 1.96 [ 1.09 , 3.54 ] | = **0.0246** |
| **Model with stepwise reverse selection** | | | |
| Gleason score | 5-6, 7, 8-10 | 2.17 [ 1.29 , 3.65 ] | = **0.0035** |
| Margin status | no tumor, positive surgical margin | 1.69 [ 0.95 , 3.02 ] | = 0.0765 |
| PTEN FISH ratio | no deletion (> 60%), deletion (≤ 60%) | 2.00 [ 1.11 , 3.60 ] | = **0.0208** |

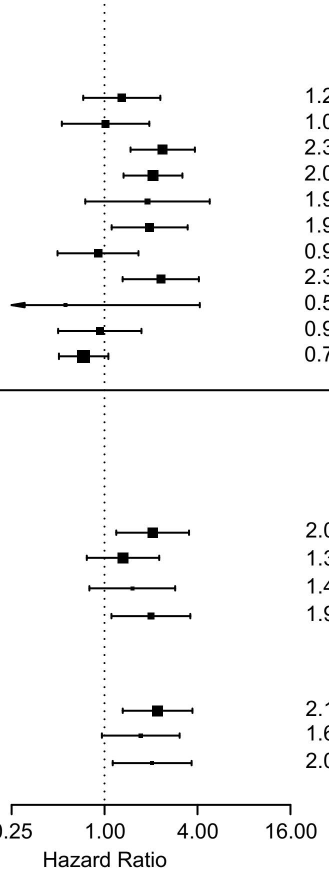


**Supplementary Figure S3.** Estimation of overall survival hazard ratios by Cox regression. The dashed vertical line was drawn at the no effect point (hazard ratio of 1.0). Horizontal lines represent a 95% confidence interval. The mid-point of the box represents the mean effect estimate and the area of the box represents the weight for each subgroup. $P < 0.05$ are marked in bold. Limit for the stepwise reverse selection procedure was $P = 0.1$.

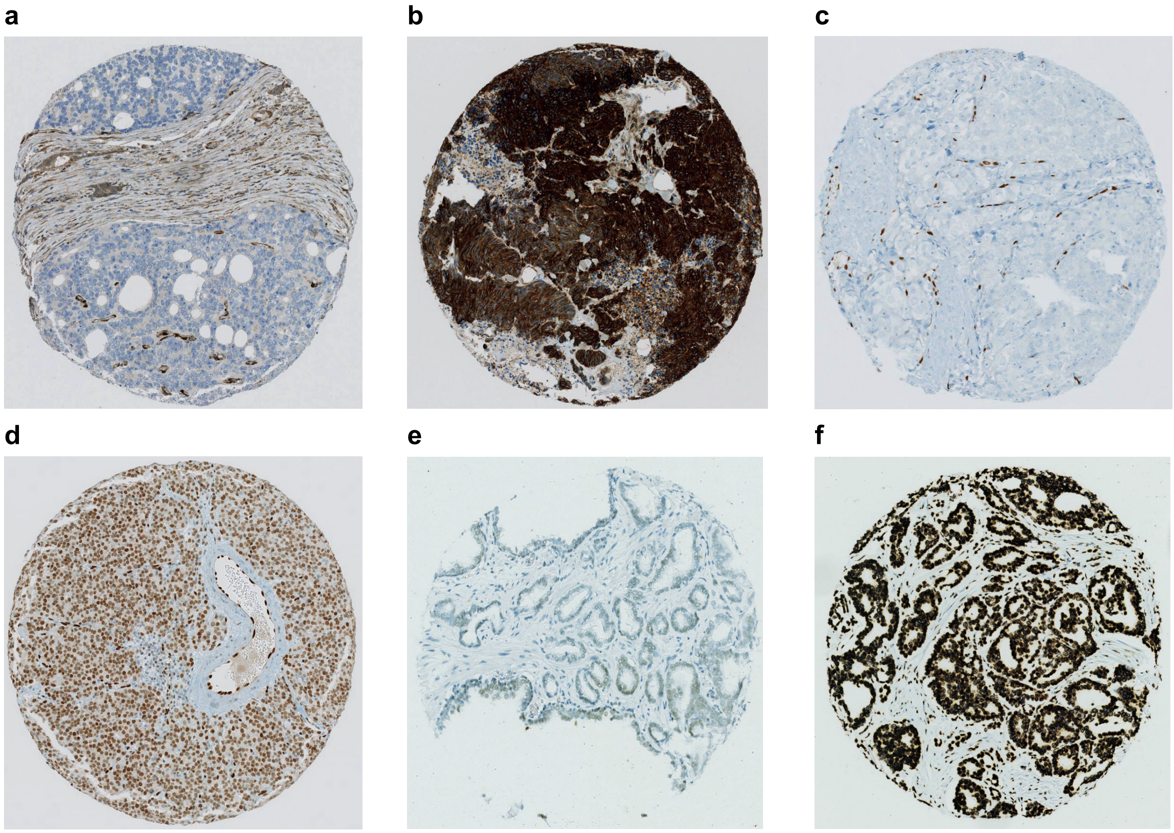


**Supplementary Figure S4.** Sample tissue cores with IHC and ALU. (**a-d**) Examples of representative tissue cores (diameter 0.6 mm) with negative (score 0) and strongly positive (score 3+) immunoreactivity for antibodies against *PTEN* and ERG. (**e**,**f**) Examples of representative tissue cores with weak and strongly positive ALU SISH as marker for DNA viability. If ALU staining is weak or negative, the DNA is not viable (no target for staining). A total of 13 cores (out of 84) were excluded from further analyses because of unviable DNA, lack of target tissue, or weak CEPs.

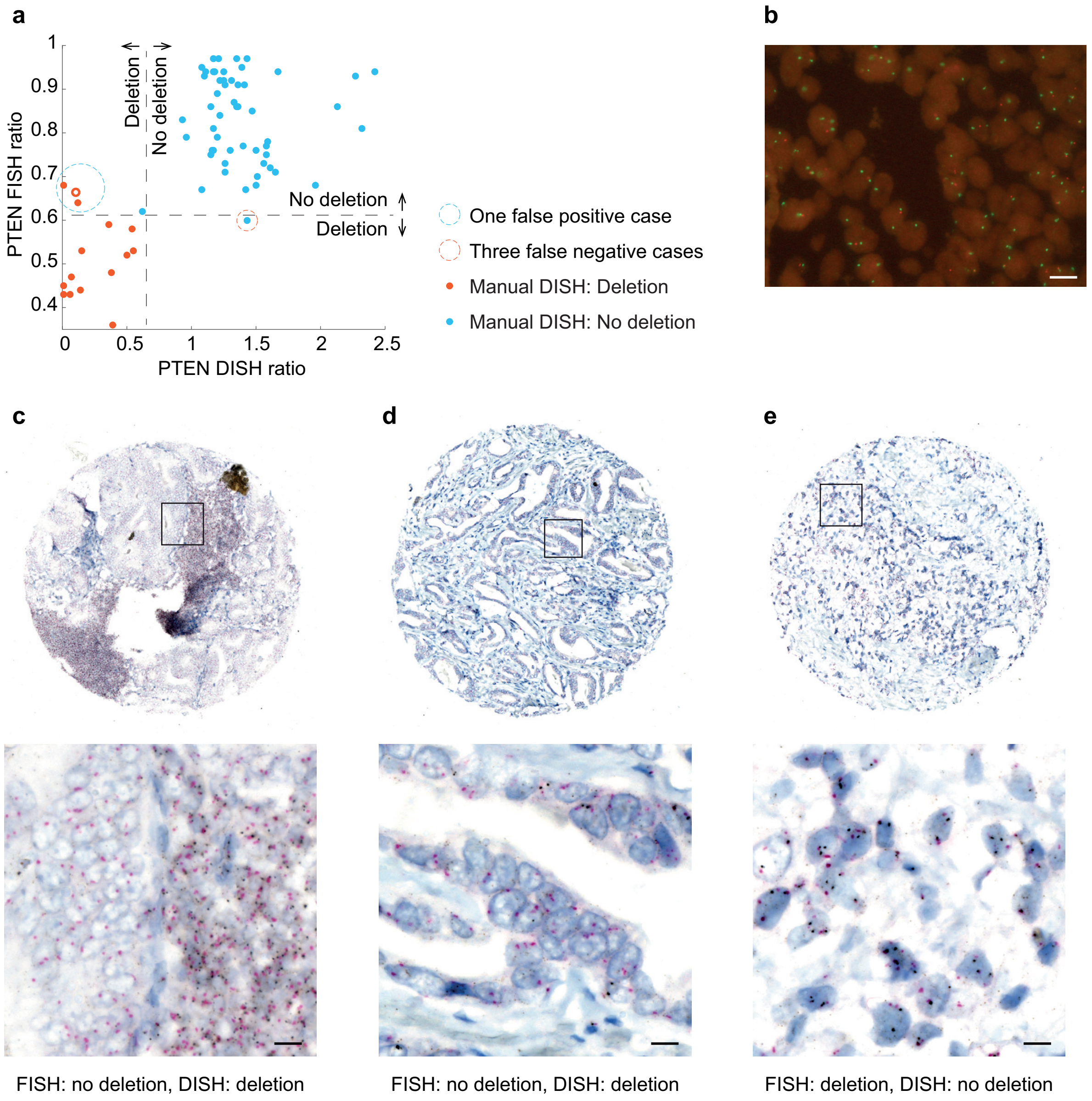


**Supplementary Figure S5.** FISH and DISH comparison. (**a**) Scatterplot of DISH ratio against FISH ratio, color-coded by DISH manual assessment. Dashed circles highlight the single false negative case (FISH: deletion; DISH: no deletion) and the three false positive cases, where the orange circle represents **Fig. 1A**. (**b**) Example of interphase FISH of prostate cancer with heterozygous *PTEN* deletion. Red, *PTEN* signals; green, CEP10 signals. Scale bar, 10 $\mu$m. (**c**,**d**) The core (diameter 0.6 mm) shows deletion by DISH, but no deletion by FISH. These two cases correspond to the two orange points circled by the blue dashed line in **a**. Scale bar, 10 $\mu$m. (**e**) DISH shows no deletion, but deletion by FISH. This case is highlighted as the single blue point circled by the orange dashed line in **a**. Scale bar, 10 $\mu$m.

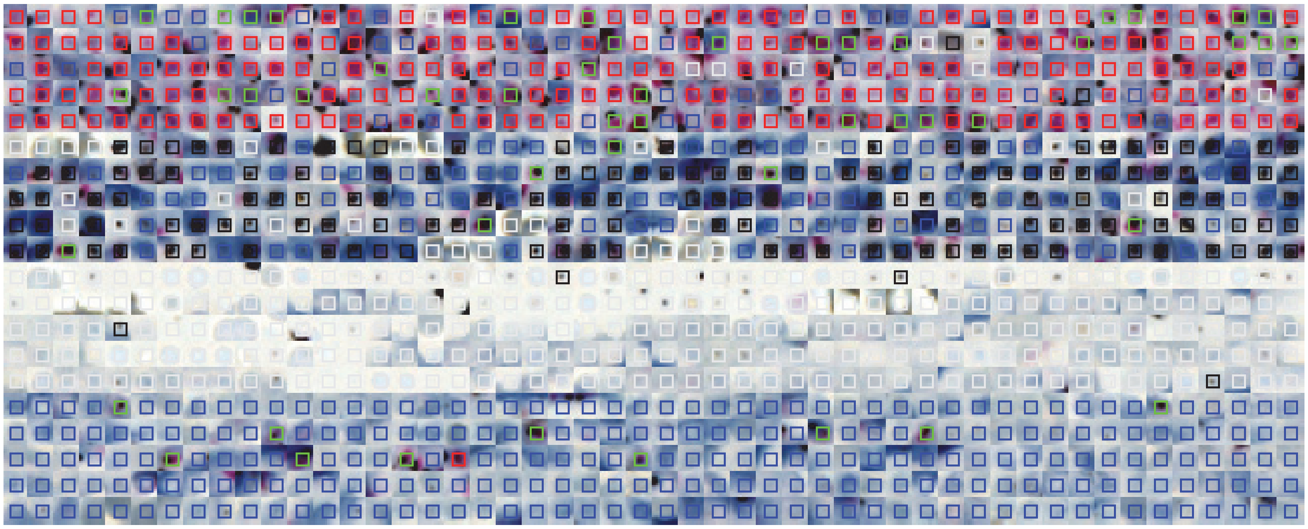

**Supplementary Figure S6.** An independent training set. A training set of 1,000 image patches from an independent prostate cancer tissue core for expert annotation, SVM training, and validation. Each image patch is centered with a roundish DISH signal of either *PTEN* (black boxes), CEP10 (red boxes), *PTEN*+CEP10 (green boxes), background noise (white boxes) or cell stain (blue boxes).

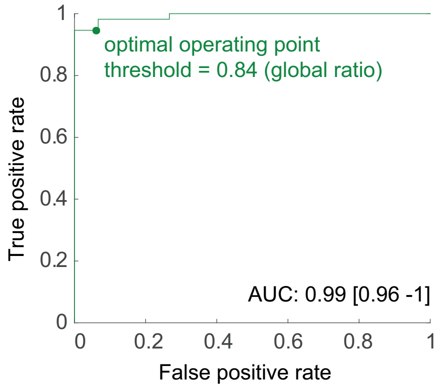


**Supplementary Figure S7.** Receiver operating characteristic (ROC) analysis. ROC analysis of global ratio using DISH manual annotation as ground truth. The optimal operating point determines the final threshold: 0.84, which were applied to (**Supplementary Table S3**). AUC stands for area under the curve.

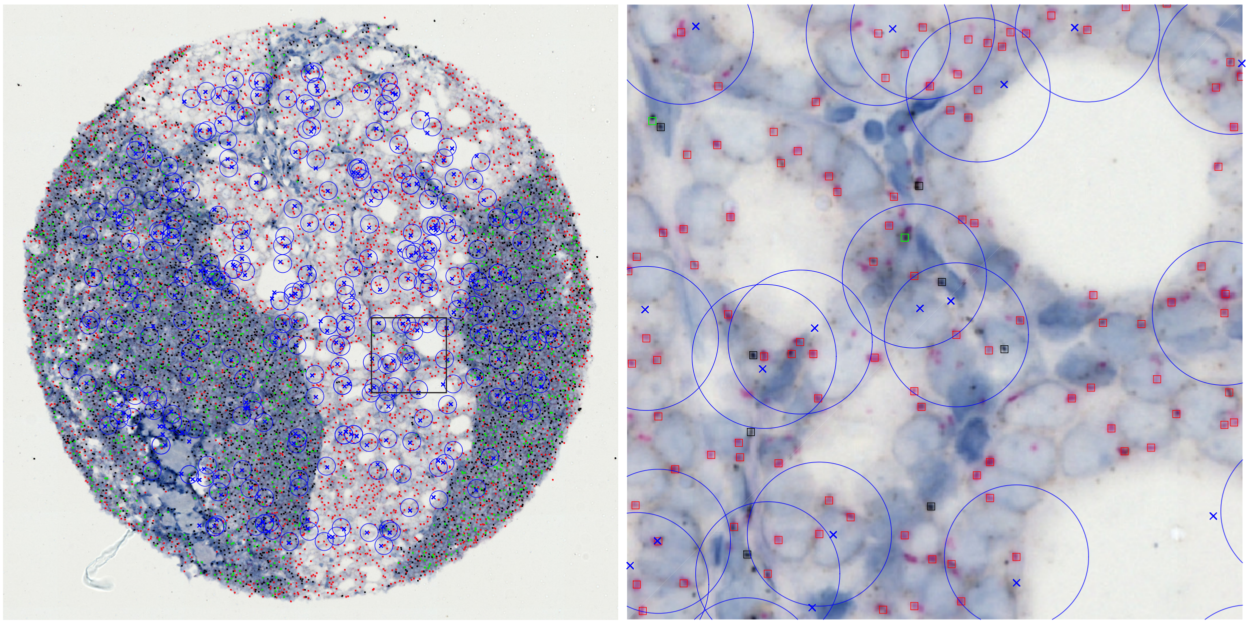

**Supplementary Figure S8.** Randomized neighborhood. The right images are the zoomed version of the left (diameter 0.6 mm), superimposed with detected points (drawn as squares, color-coded as in **Fig. 1g**) and random neighborhoods. A neighborhood is represented as a circle with a predefined radius. The center of such a circle is the CEP10 point that lies closest to a random point (in blue).

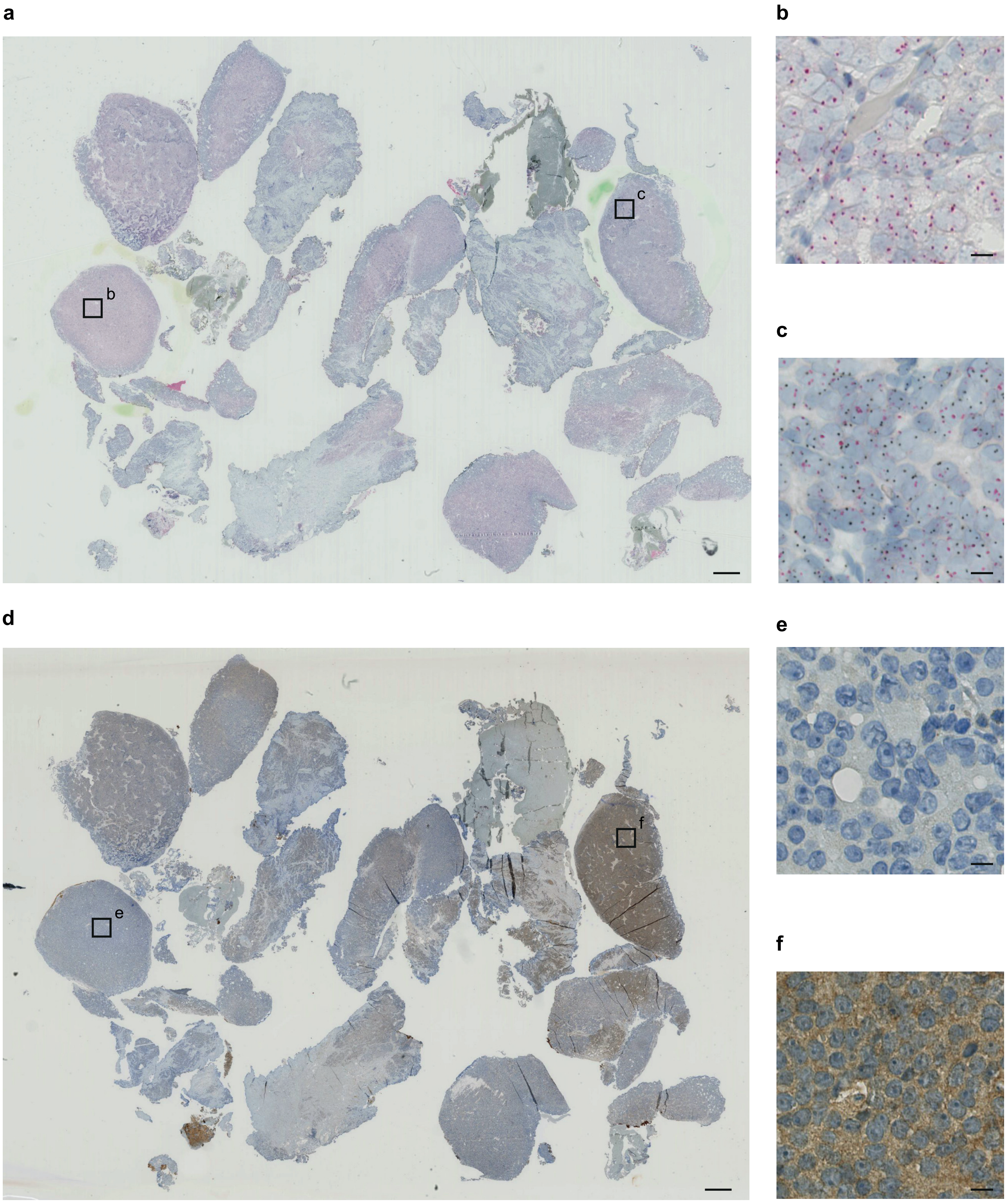


**Supplementary Figure S9.** *PTEN* DISH whole slide image analysis.(**a**) *PTEN* DISH whole slide ($108,000 \times 138,000$ pixels), scale bar 1mm. (**b**,**c**) Zoomed versions of sub areas of **a**, scale bar 10 $\mu$m. Homozygous *PTEN* deletion in **b** and no deletion in **c**. (**d**) A serial section that was immunohistochemically stained by anti-*PTEN* antibody, scale bar 1 mm. (**e**,**f**) Zoomed versions of sub areas of **d**, scale bar 10 $\mu$m. Loss of *PTEN* protein expression in **e** and cytoplasmic *PTEN* protein expression in **f**.

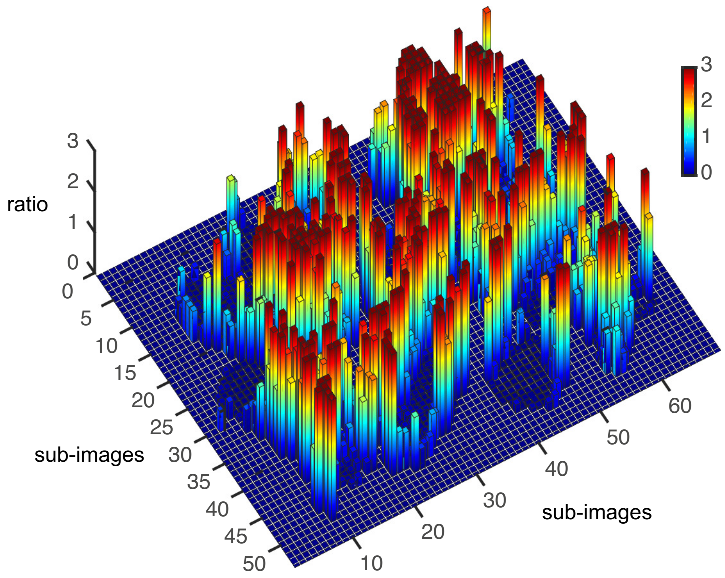


**Supplementary Figure S10.** Three-dimensional bar graph visualizing ratio distribution. The height and the color of the 3D bar graph encode the global ratio of *PTEN* to CEP10 in respective sub-images. Each sub-image has the dimension of $2000 \times 2000$ pixels.

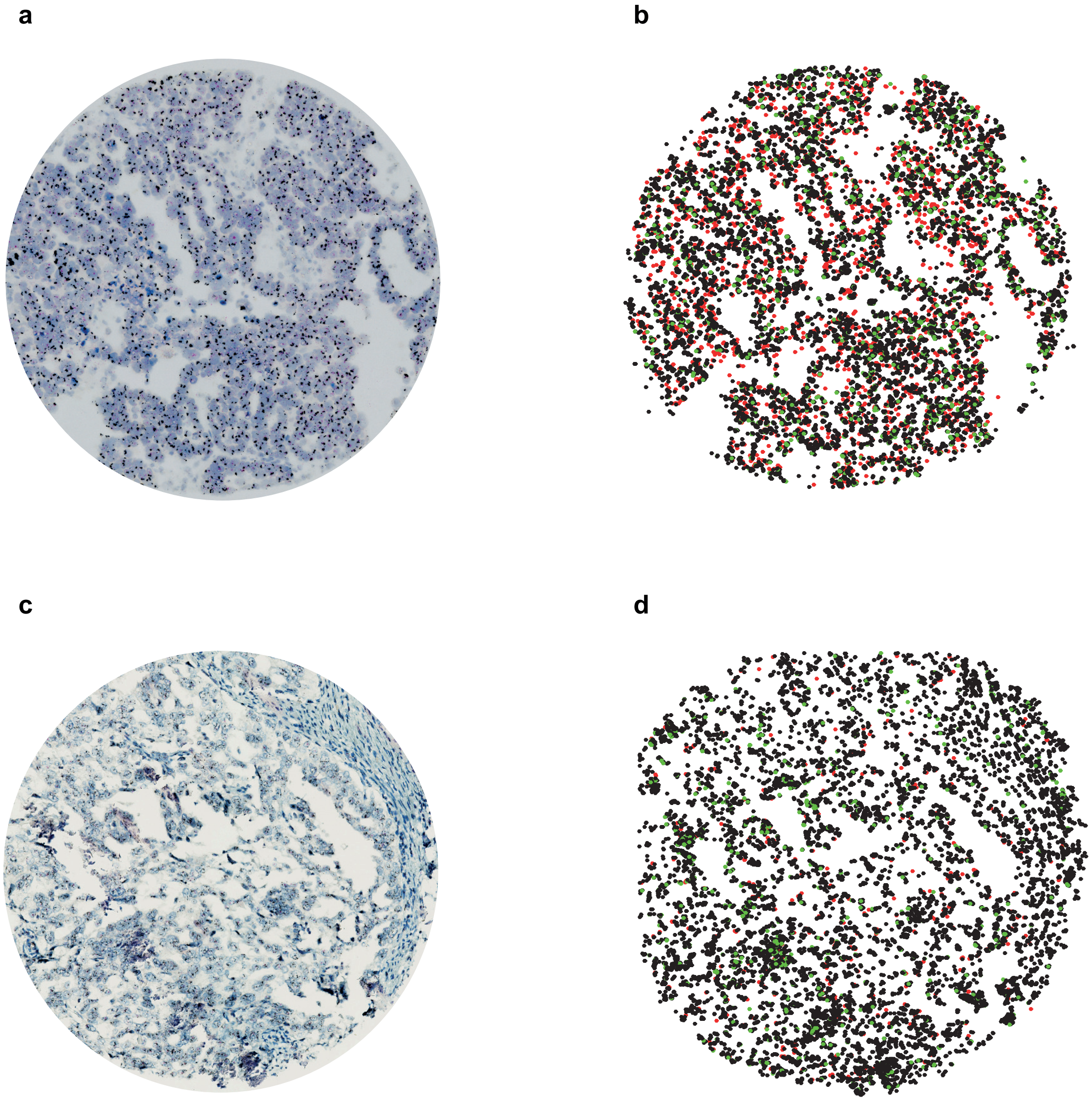


**Supplementary Figure S11.** Application of ISHProfiler to other genes. (**a**) Tissue core showing amplification of 19q12 including *CCNE1* and *URI* in ovarian cancer. (**b**) Amplification of 19q12 could be detected by the global ratio for the threshold at 2.0, matching the manual assessment of the ovarian cancer tissue core by a trained pathologist. Black signals, 19q12 gene locus; red signals, CEP19; green signals, 19q12+CEP19. (**c**) Tissue core showing amplification of 19q12 including *CCNE1* and *URI* in endometrial cancer. (**d**) Amplification of 19q12 could be detected by the ISHProfiler workflow, although the digitization was performed with a different scanner from Hamamatsu. Amplification of 19q12 could be detected by the global ratio for the threshold at 2.0, matching the manual assessment. Signals are color coded as in **b**.

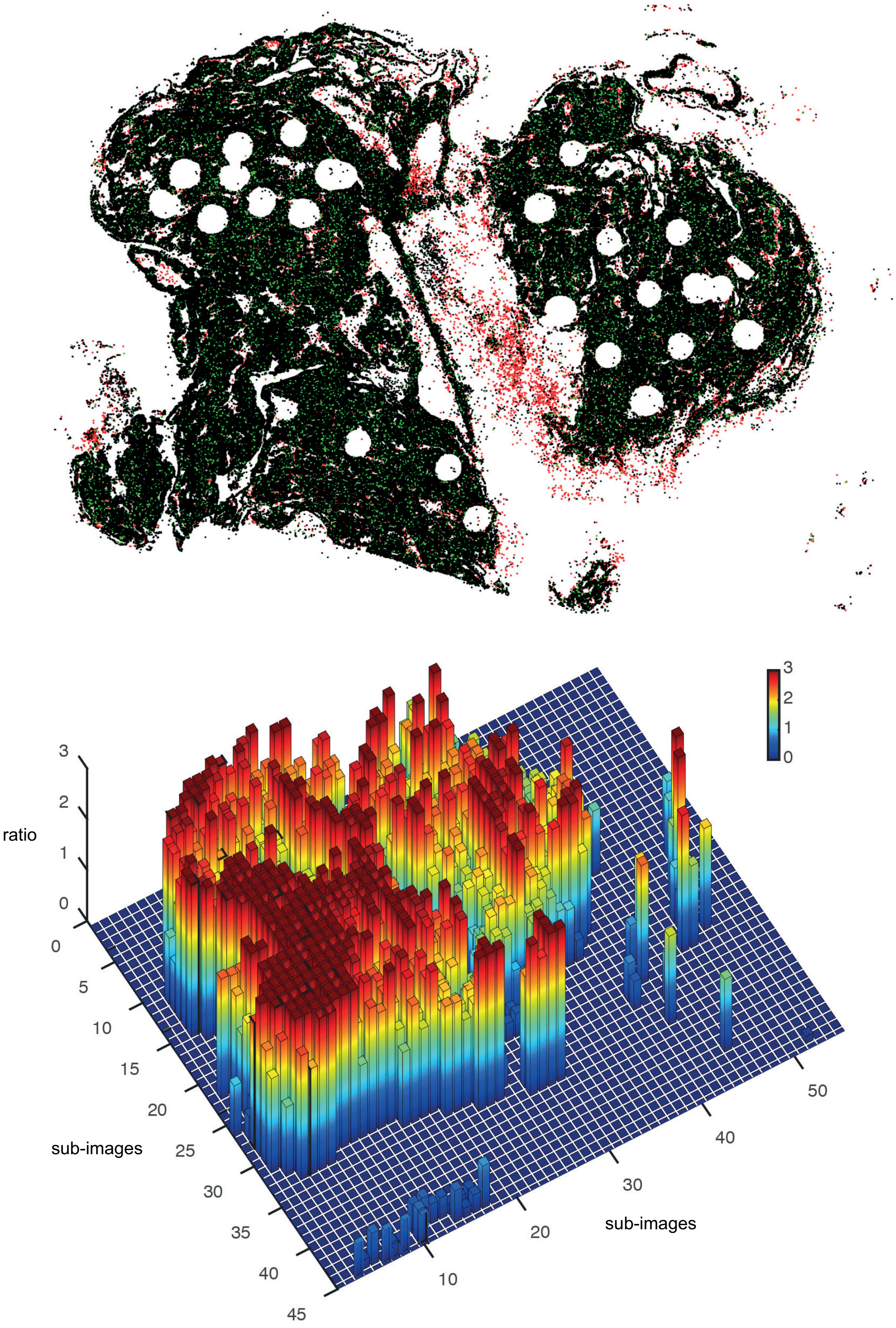


**Supplementary Figure S12.** Whole slide image analysis for 19q12 DISH. Amplification of 19q12 including *CCNE1* and *URI* in ovarian cancer could be detected on a whole slide image at the top panel, color-coded as in (**Supplementary Fig. S11b**). The height and the color of the 3D bar graph encode the global ratio of 19q12 to CEP19 in respective sub-images. Each sub-image has the dimension of $2000 \times 2000$ pixels.

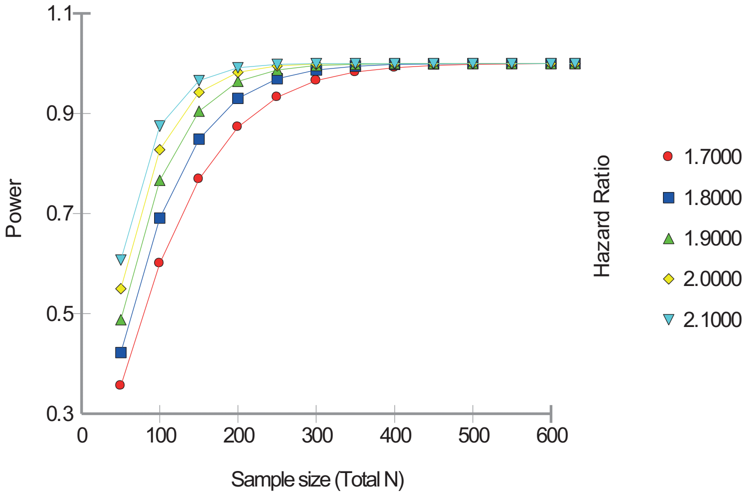


**Supplementary Figure S13.** Post hoc power analysis estimating power versus N for different hazard ratios. For instance, a two-sided log-rank test with an overall sample size of 100 subjects achieves 60.1% power at a 0.05 significance level to detect a hazard ratio of 1.70 (red dots) when the control group has a hazard ratio of 1.0. All subjects begin the study together (no accrual periods). The proportion dropping out of the both groups is 0.05.

# Supplementary Tables

Clinicopathological, immunohistochemical and molecular features of prostate cancer patients with RPE

| | |
|---|---|
| No. of patients | **339** |
| No. of patients with follow-up (%) | 298 |
| Median follow-up (months) | 95 |
| Range (months) | 0-167 |

| *Variable* | *n* | *%* |
|---|---|---|
| ***Clinicopathological characteristics*** | | |
| Age at diagnosis (median 64 years, range 46-82) | | |
| < 64 years | 145 | 42.8 |
| ≥ 64 years | 184 | 54.3 |
| unknown | 10 | 2.9 |
| Body mass index (kg/m$^2$) | | |
| ≤ 25 | 91 | 26.8 |
| > 25 | 151 | 44.5 |
| unknown | 97 | 28.6 |
| Gleason score | | |
| 5 | 7 | 2.1 |
| 6 | 46 | 13.6 |
| 7 | 198 | 58.4 |
| 8 | 24 | 7.1 |
| 9 | 59 | 17.4 |
| 10 | 3 | 0.9 |
| unknown | 2 | 0.6 |
| Tumor stage (pT) | | |
| pT2a | 37 | 10.5 |
| pT2b | 4 | 1.2 |
| pT2c | 170 | 50.1 |
| pT3a | 56 | 16.5 |
| pT3b | 56 | 16.5 |
| pT4 | 13 | 3.8 |
| unknown | 3 | 0.9 |
| Nodal status (pN) | | |
| pN0 | 266 | 78.5 |
| pN1 | 17 | 5.0 |
| unknown | 56 | 16.5 |
| Surgical margins | | |
| negative | 217 | 64.0 |
| positive | 115 | 33.9 |
| unknown | 7 | 2.1 |
| PSA at diagnosis (ng/µl) | | |
| < 10 | 131 | 38.6 |
| ≥ 10 | 165 | 48.7 |
| unknown | 43 | 12.7 |

| *Variable* | *n* | *%* |
|---|---|---|
| ***Immunohistochemical data*** | | |
| Cytoplasmic PTEN immunoreactivity (intensity) | | |
| negative | 119 | 35.1 |
| score 1+ | 95 | 28.0 |
| score 2+ | 96 | 28.3 |
| score 3+ | 6 | 1.8 |
| unknown | 23 | 6.8 |
| Nuclear ERG immunoreactivity (intensity) | | |
| negative | 156 | 46.0 |
| score 1+ | 73 | 21.5 |
| score 2+ | 58 | 17.1 |
| score 3+ | 36 | 10.6 |
| unknown | 16 | 4.7 |
| Ki-67 (MIB1) immunoreactivity (%) | | |
| < 5% | 310 | 91.4 |
| ≥ 5% | 27 | 8.0 |
| unknown | 2 | 0.6 |
| | | |
| ***Sequencing data*** | | |
| *SPOP* mutation | | |
| wild-type | 151 | 44.5 |
| mutation | 13 | 3.8 |
| unknown | 175 | 51.6 |
| | | |
| ***Fluorescence in situ hybridization*** | | |
| *ERG* FISH (break apart assay) | | |
| normal | 124 | 36.6 |
| translocation | 50 | 14.7 |
| translocation through deletion | 89 | 26.3 |
| mixture (normal/translocation) | 4 | 1.2 |
| unknown | 72 | 20.9 |
| *PTEN* FISH (ratio) | | |
| no deletion (ratio > 0.6) | 261 | 77.0 |
| deletion (ratio ≤ 0.6) | 78 | 23.0 |
| unknown | 0 | 0.0 |
| *PTEN* FISH (% aberrant nuclei) | | |
| no deletion (% < 0.6) | 265 | 78.2 |
| hemizygous deletion (% ≥ 0.6) | 32 | 9.4 |
| homozygous deletion (% ≥ 0.6) | 42 | 12.4 |
| unknown | 0 | 0.0 |

**Supplementary Table S1.** Clinicopathological and immunohistochemical features of patients with prostate cancer receiving RPE.

| | | PTEN FISH ratio (dichotomous 60%) | | |
|---|---|---|---|---|
| | | PTEN Deletion | No Deletion | Total |
| PTEN DISH ratio | PTEN Deletion | 12 | 3 | 15 |
| (dichotomous 60%) | No Deletion | 1 | 55 | 56 |
| Total | | 13 | 58 | 71 |
| Accuracy: 94.4 | | Sensitivity: 92.3% | Specificity: 94.8% | |

**Supplementary Table S2.** Sensitivity and specificity for *PTEN* FISH and DISH.

| | | PTEN global ratio (optimal threshold 84%) | | |
|---|---|---|---|---|
| | | PTEN Deletion | No Deletion | Total |
| PTEN DISH ratio | PTEN Deletion | 14 | 1 | 15 |
| (dichotomous 60%) | No Deletion | 1 | 55 | 56 |
| Total | | 15 | 56 | 71 |
| Accuracy: 97.2 | | Sensitivity: 93.3% | Specificity: 98.2% | |

**Supplementary Table S3.** Sensitivity and specificity for the *PTEN* DISH ratio and the global ratio. The global ratio was dichotomized by the optimal threshold of 0.84, determined by the ROC analysis in (**Supplementary Fig. S7**).
